# Highly reducible polyoxometalate–Dy(iii) SMM hybrid materials with exceptional charge stability

**DOI:** 10.1039/d5sc07950k

**Published:** 2025-11-18

**Authors:** Ethan Lowe, Mathieu Rouzières, Sarah K. Dugmore, Christopher Kelly, Claire Wilson, Angelos B. Canaj, Rodolphe Clérac, Mark Murrie

**Affiliations:** a School of Chemistry, University of Glasgow University Avenue Glasgow G12 8QQ UK mark.murrie@glasgow.ac.uk; b Univ. Bordeaux, CNRS, CRPP, UMR 5031 33600 Pessac France rodolphe.clerac@u-bordeaux.fr; c The Henry Royce Institute, The University of Manchester Royce Hub Building Manchester M13 9PL UK

## Abstract

Lanthanide single-molecule magnets (SMMs) continue to draw attention as potential building blocks for ultra-dense data storage devices due to their bistable magnetic ground states and pronounced magnetic anisotropy. To realise this potential, however, a deeper understanding of how molecular magnetic memory responds to structural and environmental perturbations is critical. One essential criterion is the retention of magnetic bistability in the presence of nearby charge or charge fluctuations. Air-stable Dy(iii) SMMs with pseudo-*D*_5h_ symmetry are known to exhibit extremely slow magnetic relaxation, attributed to a strong axial crystal field and symmetry-imposed suppression of quantum tunnelling of magnetisation (QTM). Here we report a new high-performance, hybrid compound, [Dy(H_2_O)_5_(Cy_3_PO)_2_][Mo_12_PO_40_]·2(Cy_3_PO)·4THF·2H_2_O·Et_2_O (1), incorporating the bulky polyoxometalate [Mo_12_PO_40_]^3−^ in the second coordination sphere. Upon exposure to UV light or X-rays, partial reduction of Mo(vi) to Mo(v) (*ca*. 3%) yields 1_Red_, a hybrid material that demonstrates enhanced magnetic blocking, evidenced by increased *T*_B(Hyst)_ and *T*_IRREV_ relative to 1. Importantly, we introduce a dilution strategy using an optically dilute, diamagnetic KBr matrix to enhance Mo reduction. This approach boosts Mo(v) content to *ca*. 30% in 1_Red_@KBr while preserving the slow relaxation dynamics of the Dy(iii) complex. These results highlight the magnetic resilience of the [Dy(H_2_O)_5_(Cy_3_PO)_2_]^3+^ motif in charged environments and establish a basis for exploring magneto-optical and magneto-electric behaviours in SMM hybrid materials.

## Abstract 2

High performance polyoxometalate–Dy(iii) SMM hybrid compounds exhibiting photoreduction.

## Graphical abstract

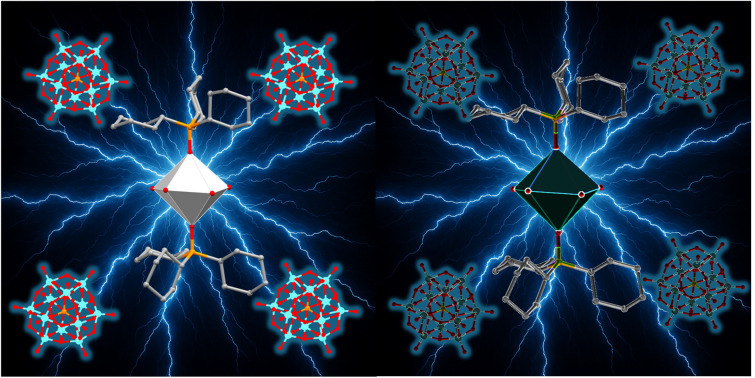

## Introduction

The potential to design molecular systems with a bistable ground state and a large energy barrier to the reversal of magnetisation (*U*_eff_) makes single-molecule magnets (SMMs) highly attractive prospects for applications in data storage, molecular spintronics and as qubits.^1–3^ In order to realise these applications, extensive work has been conducted to increase the working temperatures to that of liquid nitrogen.^4–7^ This has been made possible not only by the design of complexes exhibiting large *U*_eff_ barriers, but also by the suppression of through-barrier relaxation mechanisms such as Raman and quantum tunnelling of the magnetisation (QTM).^8–10^ Following the vast number of ‘high-performance’ single-molecule magnets exhibiting *U*_eff_ barriers greater than 500 K, significant attention has turned to the design of multi-functional SMMs.^11,12^ These species combine the SMM slow magnetic relaxation with a plethora of interesting properties such as luminescence, conductivity, chirality and photochromism that are afforded by the synthetic tuneability of molecular species.^13–16^ Here, both synthetic flexibility and structural stability are advantageous properties to allow for the incorporation of new functional groups and molecules that can tune the interplay between these phenomena.

Previously, we showed that the anion substitution of three triflate anions for a large trianionic α-Keggin polyoxotungstate engenders an increased *U*_eff_ barrier in the pseudo-*D*_5h_ compound [Dy(H_2_O)_5_(Cy_3_PO)_2_][W_12_PO_40_]·2(Cy_3_PO)·5THF·H_2_O [Cy_3_PO = tricyclohexylphosphine oxide].^17^ The persistence of a large *U*_eff_ is extremely rare in such hybrid Dy(iii) polyoxometalate (POM) compounds, with this being the only example where incorporation of a polyoxometalate into a pseudo-*D*_5h_ system results in an increased *U*_eff_ barrier.^18–21^ This is in part due to the plethora of coordination modes exhibited by many W-based POMs, making coordination of polyoxometalates extremely common.^21*b*,*c*^ Unwanted ligand substitution has also been observed previously in the [Dy(H_2_O)_*x*_(R_3_PO)_*y*_]^3+^ unit upon POM integration, resulting in their equatorial coordination.^18^ This increase in equatorial charge results in the introduction of transverse crystal field parameters, which significantly reduce the size of the *U*_eff_ barrier and introduce fast magnetic relaxation through QTM.

The functionalisation of POMs has been widely studied with incorporation of transition metals, lanthanide ions and heteroatoms leading to a diverse range of properties.^22–25^ Importantly, POMs have been shown to exhibit a wealth of interesting redox chemistry, photochromism, electrical conductivity and catalytic activity.^26–29^ The plethora of fascinating properties that POMs possess make them exceptional candidates in the design of multifunctional SMMs^30^ for sensing and switchable materials. The stability of POMs upon reduction and the ability to act as redox reservoirs may modulate or switch SMM spin relaxation dynamics when in close proximity. Furthermore, the fundamental importance of the second coordination sphere in Ln(iii) SMMs has been studied,^30*b*–*d*^ particularly in pseudo-*D*_5h_ systems,^4,31,32^ and changes in electron density around the Ln(iii) centre are expected to have significant effects on multiple relaxation mechanisms. For an oblate Dy(iii) ion, the presence of additional charge in the equatorial region is expected to both decrease *U*_eff_ and increase the rate of QTM.^33,34^ Importantly, in this pseudo-*D*_5h_ system incorporation of the Mo-based polyoxometalate is also expected to generate a significant change in the hydrogen bonding which is expected to modulate the spin–phonon coupling between the Dy(iii) centre and the equatorial H_2_O ligands, thus modulating relaxation of the Dy(iii) magnetic moment through the Raman relaxation mechanism.^34*b*^

Tungstate-based polyoxometalate–SMM compounds have been studied in part due to their robust stability, vast array of architectures and rigid W–O based frameworks.^21*b*,34*c*,34*d*^ However, [Mo^VI^_12_PO_40_]^3−^ possesses a reduced redox potential compared to the isostructural [W^VI^_12_PO_40_]^3−^, displaying vastly different physical properties to its tungstate analogue.^35^ This easier reduction allows for the incorporation of delocalised electrons into a polyoxometalate–SMM hybrid material. This is expected to affect the slow magnetic relaxation of the Dy(iii) centre by a number of possible mechanisms: (i) the unpaired electrons on the polyoxometalate will possess a magnetic moment, which may couple to the Dy(iii) centre, modulating its magnetic properties; (ii) the reduction will result in an increased negative charge/electron density in the equatorial region, introducing stronger transverse crystal field parameters, which may generate faster QTM and a smaller *U*_eff_ energy barrier; (iii) the change in charge on the polyoxometalate is expected to alter any hydrogen bonding network that exists within the crystal structure, this may result in stronger hydrogen bonding and a more rigid structure, suppressing the spin–phonon coupling and Raman relaxation.^34*b*^ It should also be noted that, in the reduction of Mo-based POMs, a significant lengthening of the M=O bonds is observed, which can be expected to introduce a further change in vibrational modes that may also affect relaxation *via* a Raman mechanism.^36^

With this in mind, we have investigated the incorporation and subsequent reduction of the isostructural α-Keggin ion [Mo^VI^_12_PO_40_]^3−^ into [Dy(H_2_O)_5_(Cy_3_PO)_2_](CF_3_SO_3_)_3_·2(Cy_3_PO) *via* the anion substitution reaction highlighted in our previous work.^17^ An enhanced reduction of the polyoxometalate can be obtained by dilution into an optically dilute diamagnetic matrix or irradiation with X-rays, resulting in an optically tuneable polyoxometalate based SMM hybrid.

## Results and discussion

Compound 1 was synthesised using a [Dy(H_2_O)_5_(Cy_3_PO)_2_](CF_3_SO_3_)_3_·2(Cy_3_PO) (P1) precursor in an anion substitution reaction. A THF solution of P1 along with H_3_[Mo_12_PO_40_], dissolved in a small amount of water, was heated and after workup, vapor diffusion using Et_2_O yielded yellow block crystals of [Dy(H_2_O)_5_(Cy_3_PO)_2_][Mo_12_PO_40_]·2(Cy_3_PO)·4THF·2H_2_O·Et_2_O (1) where the three triflate anions in P1 are replaced by a bulky trianionic polyoxometalate (POM) anion. Upon removal of the yellow crystals of 1 from the fridge and under exposure to light the crystals rapidly change in colour to green, before turning blue over longer periods of time.

### Crystal structure

**Fig. 1:**
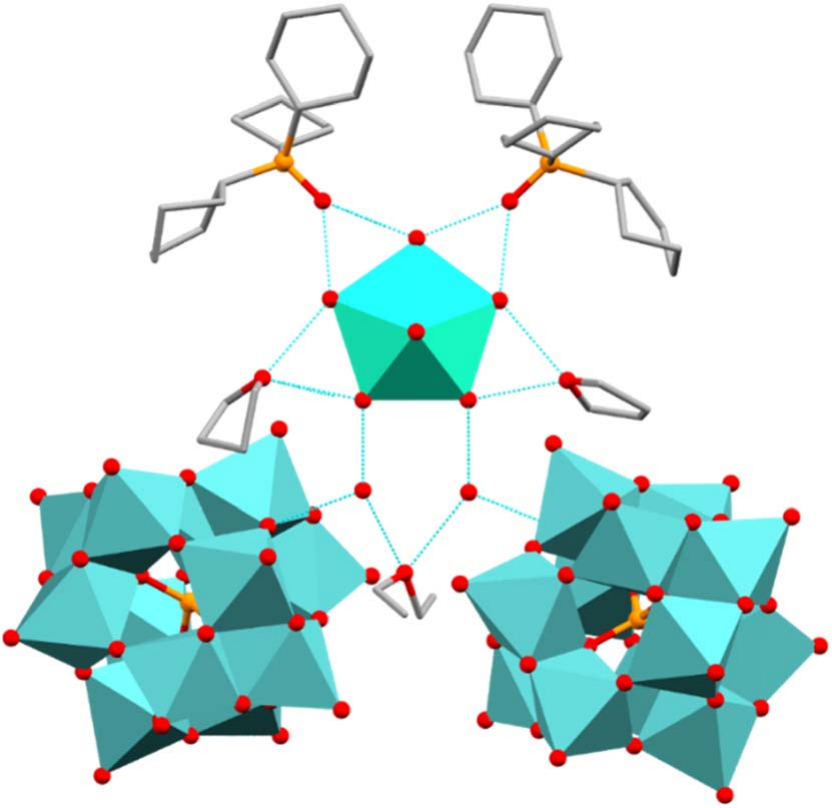
The hydrogen-bonding network in 1 with axial {Cy_3_PO} units and H atoms omitted for clarity (only one POM anion is present within the asymmetric unit). C, grey; Dy, cyan; O, red; P, orange; Mo, light blue.

Compound 1 crystallises in the monoclinic space group *Cc* (Table S1). The asymmetric unit contains a [Dy(H_2_O)_5_(Cy_3_PO)_2_]^3+^ cation, one [Mo_12_PO_40_]^3−^ anion, four THF, two H_2_O, two Cy_3_PO and one Et_2_O molecules (Fig. S1). The Dy(iii) ion exhibits a distorted pentagonal bipyramidal geometry (∼*D*_5h_) as confirmed by continuous shape measures analysis, CShMs = 0.217.^37^ Short average axial Dy–O bond lengths of 2.20(2) Å and longer equatorial Dy–O bond lengths averaging 2.36(2) Å are observed in 1. The axial O–Dy–O angle of 178.9(5)° and average equatorial O–Dy–O angles of 72.1(6)° are very close to the ideal angles of 180° and 72° in a perfect pentagonal bipyramid. The second coordination sphere consists of intricate hydrogen bonding networks, where the [Dy(H_2_O)_5_(Cy_3_PO)_2_]^3+^ unit is hydrogen bonded to two THF, two H_2_O and two co-crystallised Cy_3_PO molecules in 1. The two H_2_O molecules are then hydrogen bonded to one Et_2_O molecule and two [Mo^IV^_12_PO_40_]^3−^ anions (Fig. 1). The O⋯O distances between equatorial H_2_O ligands and the second coordination sphere are 2.66(2), 2.67(3), 2.67(2) and 2.60(2) Å (O⋯OPCy_3_), 2.63(2), 2.69(2) (O⋯OH_2_) and 2.80(2), 2.70(3), 2.78(2), 2.76(2) (O···OC_4_H_8_). The centre of the POM anion lies 10.7 Å away from the Dy(iii) centre and 27.5° out of the {Dy(H_2_O)_5_} equatorial plane (Fig. S1). Whilst the POM lies a significant distance away from the Dy(iii) centre, this non 90° Dy(iii)–POM angle is expected to introduce transverse crystal field parameters which may contribute towards an increased rate of relaxation through QTM and a reduced *U*_eff_ barrier, similar distances and angles were observed in our previous work.^17^ The shortest intermolecular Dy⋯Dy distance is 15.6 Å, which can be expected to reduce intermolecular dipole–dipole interactions (Fig. S2).^38^ Single crystals of 1_Red_ (*vide infra*) were obtained by leaving yellow single crystals of 1 in indirect sunlight for one week. Compound 1_Red_ also crystallises in the monoclinic space group *Cc* (Table S1) with extremely similar lattice parameters to 1 [*a* = 24.597(2), *b* = 19.211(1), *c* = 29.522(2) *vs. a* =24.6687(9), *b* =19.2311(9), *c* =29.303(2)]. The Dy(iii) ion exhibits a similar distorted pentagonal bipyramidal geometry (∼*D*_5h_) to 1 and possesses extremely similar average axial Dy–O bond lengths of 2.20(2) Å and equatorial Dy–O bond lengths averaging 2.36(1) Å.

The centre of the POM anion lies approximately 10.4 Å away from the Dy(iii) centre and 26.6° out of the {Dy(H_2_O)_5_} equatorial plane and the shortest intermolecular Dy⋯Dy distance is 15.6 Å. As such the SIM and POM units in 1_Red_ show a near identical similarity to 1. Some possible changes to the solvent molecules located within the second coordination sphere may be indicated by the need for a solvent mask (see SI).

### Photochromic behaviour

Compound 1 was ground in a pestle and mortar for 30 minutes and left in indirect sunlight for one week. This resulted in a blue homogeneous powder denoted as 1_Red_ herein. Importantly, no significant change between 1 and 1_Red_ was observed within the single-crystal or powder X-ray diffraction, IR, or elemental analysis (Tables S1, S2 and Fig. S3–S6).

**Fig. 2:**
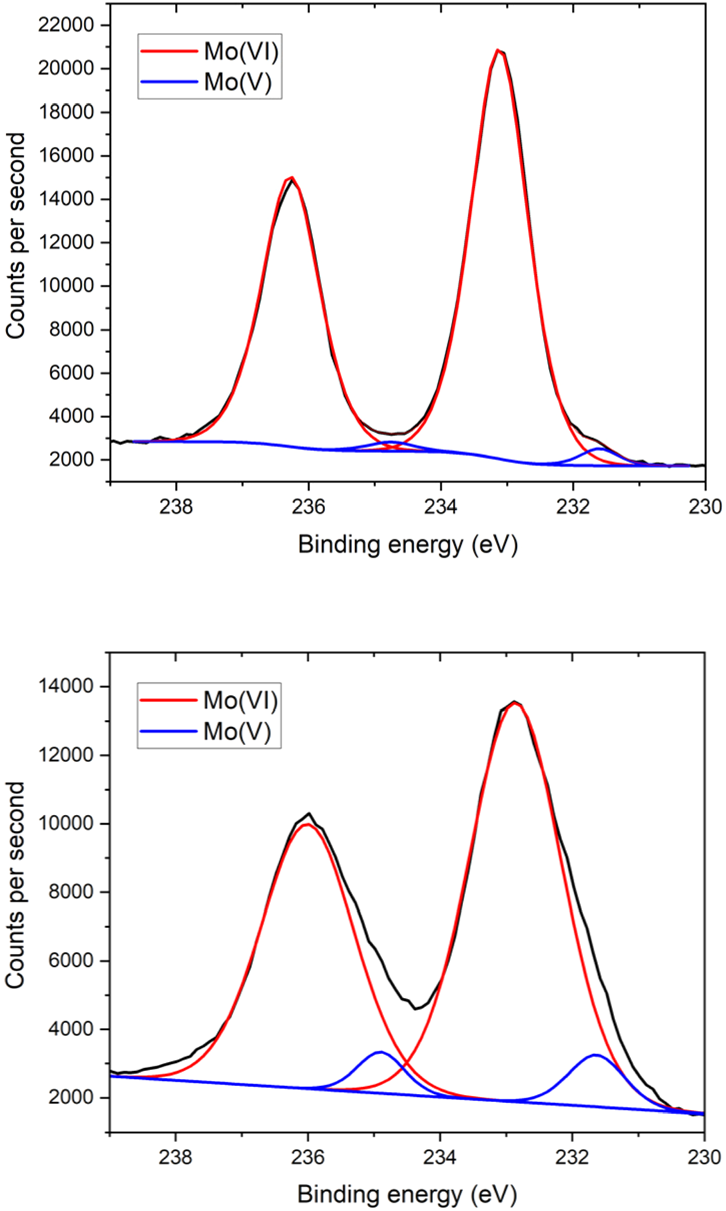
The initial Mo 3d XPS spectra of compound 1 (top) and 1_Red_ (bottom), where the black line represents the counts per second, the red line represents the fit to Mo(vi) and the blue line is the fit to Mo(v).

The single crystals and powder samples of 1_Red_ undergo a slight loss of crystallinity over time, resulting in a slight broadening of the PXRD powder pattern (Fig. S5) and a reduced completeness in the single-crystal X-ray diffraction data compared to 1 (Table S1). To quantify the change in colour in 1_Red_, X-ray photoelectron spectroscopy measurements were conducted (Fig. S7) to probe for the presence of Mo(v) in 1 and 1_Red_. Ground samples of 1 and 1_Red_ were compressed into flat discs before measurement. For 1, two different Mo(vi) 3d signals are observed at binding energies of approximately 233 and 236 eV, which are characteristic of the 3d_5/2_ and 3d_3/2_ energy levels in Mo(vi).^39,40^ These are accompanied by two much smaller Mo(v) 3d signals at 231.5 and 234.5 eV (Fig. 2). Decomposition of the 3d doublets results in a 1 : 36 ratio of Mo(v) to Mo(vi), indicating only a very small proportion of the sample is reduced. We attribute this minimal reduction within 1 to radiation damage to the sample (*vide infra*) sustained within the 60 seconds measurement time required to obtain the spectra. Compound 1_Red_ also possesses two different Mo(vi) 3d signals at binding energies of approximately 233 and 236 eV, characteristic of the 3d_5/2_ and 3d_3/2_ energy levels in Mo(vi).^39,40^ These are also accompanied by two smaller Mo(v) 3d signals at 231.5 and 235 eV (Fig. 2). Decomposition of the 3d doublets results in a 1 : 12.7 ratio of Mo(v) to Mo(vi), indicating that roughly one of the Mo(vi) centres is reduced per polyoxometalate, which produces the dark blue colour (see mechanistic insights section below).^41^

**Fig. 3:**
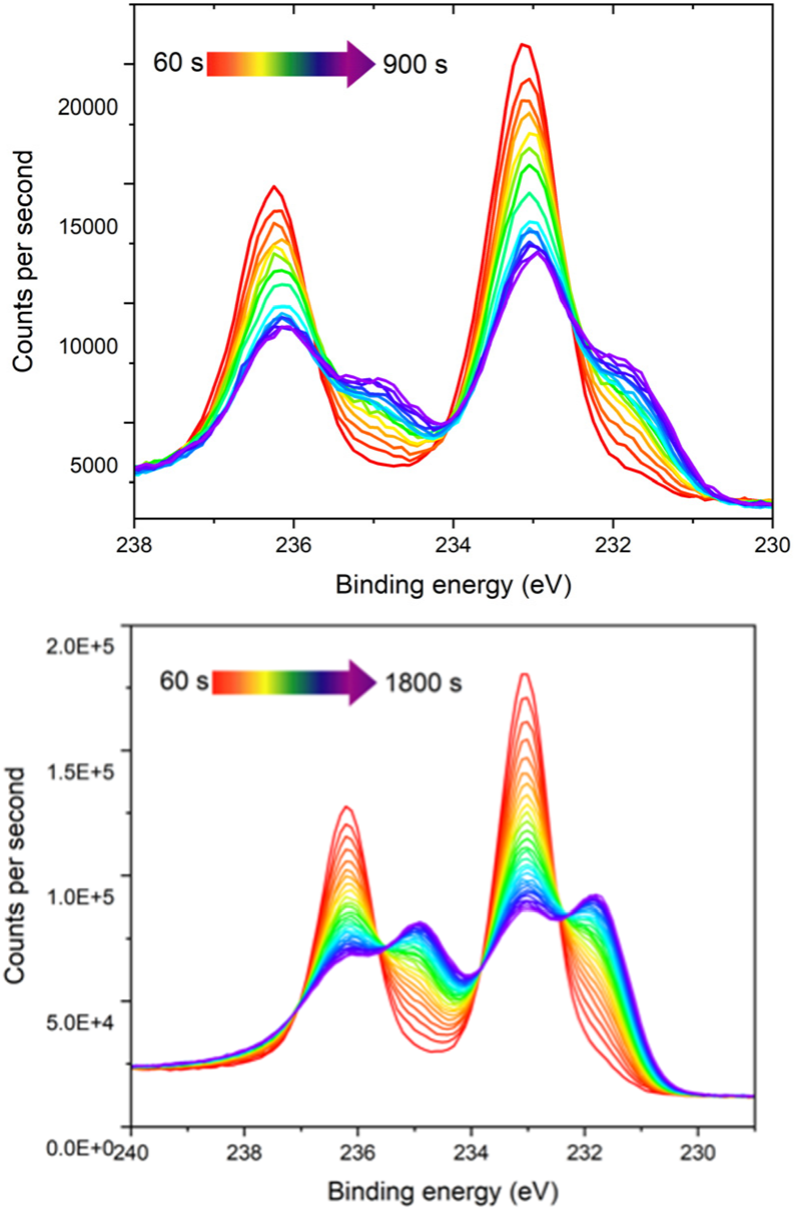
The time-resolved Mo 3d XPS spectra of compound 1 (top), and 1_Red_ (bottom) from 60 second measurement time (red) to 900 s (purple) (top) and 1800 s (purple) (bottom).

Due to the observation of an enhanced colour change upon irradiation with X-rays (Fig. S8), time resolved XPS measurements were conducted at 60 second intervals on samples of 1 and 1_Red_. A clear decrease in the intensity of the Mo(vi) and increase in the Mo(v) 3d peaks is observed over time, with a change in the Mo(v) : Mo(vi) ratio from 1 : 36 at 60 s to 1 : 2.9 at 900 s in 1 and from 1 : 12.7 at 60 s to 1 : 1.67 at 1800 s in 1_Red_ (Fig. 3). Reduction of Mo(vi)-based materials is not uncommon during XPS measurements and has been well documented.^42–44^ The reduction of the sample is highly localised (Fig. S8). Fitting of the reduction to an exponential growth and extrapolation back to zero seconds yields an initial Mo(v) : Mo(vi) ratio of 1 : 32.3 in 1_Red_ (Fig. S9), indicating only a very small amount of the sample is reduced before characterisation takes place. This equates to roughly 3% Mo(v) and ‘0.36’ extra electrons per polyoxometalate in the reduced sample 1_Red_. In contrast, extrapolation to 0 s for compound 1 results in approximately 0% Mo(v), as expected for a pristine sample of 1.

### Solid-state optical reflectivity

**Fig. 4:**
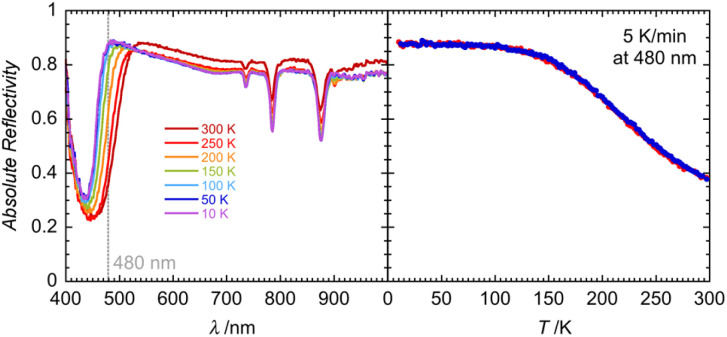
Left: temperature dependence (between 300 K and 10 K) of the optical reflectivity spectra of 1 between 400 and 1000 nm. A spectroscopic white light of 0.08 mW cm^−2^ has been used for these measurements. Right: thermal variation of the 480 nm optical reflectivity signal recorded at a scan rate of 5 K min^−1^ when cooling (blue trace) and heating (red trace) in the dark.

**Fig. 5:**
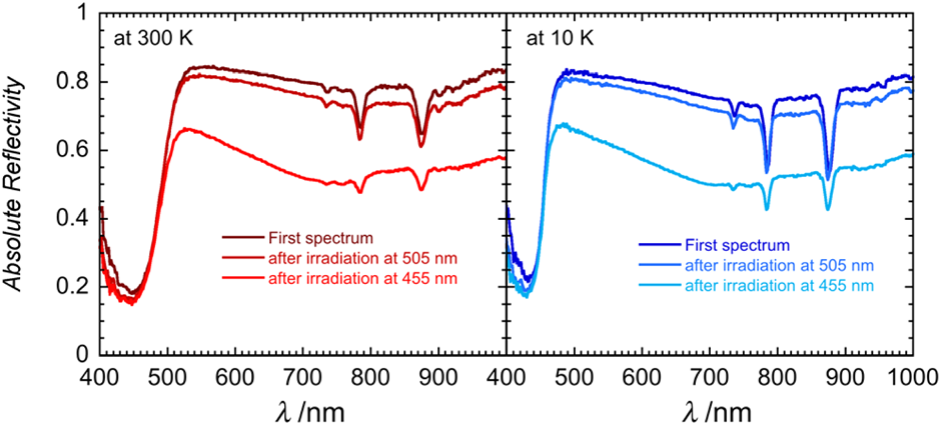
Optical reflectivity spectra of 1 between 400 and 1000 nm at 300 K (left) and at 10 K (right) showing the irreversible photosensitivity at 505 and 455 nm for comparison (10 minutes, at 16 mW cm^−2^). A spectroscopic white light of 0.08 mW cm^−2^ has been used for these measurements.

Solid-state optical reflectivity of 1 was studied as a function of temperature to attempt to rationalise the cause of the reduction (Fig. 4, 5 and S10–S13). Compound 1 exhibits three narrow absorption bands at 736, 786 and 879 nm which are attributed to Dy(iii) f–f transitions from the ^6^H_15/2_ ground state to ^6^F_3/2,_^6^F_5/2,_^6^F_7/2_ excited state multiplets, respectively.^45,46^ These bands increase in intensity as the temperature is decreased. A broad temperature dependent absorption band at 450 nm is associated with a ligand-to-metal charge transfer (LMCT) between POM bridging oxygen atoms and the Mo(vi) centres.

This traditionally occurs around 320 nm (ref. 47 and 48) in phosphomolybdic acid but is known to shift drastically depending on the coordination environment and has been observed around 450 nm in similar lanthanide-POM compounds.^21,49^ The temperature dependence of the spectra at 480 nm is clearly reversible, as shown in Fig. 4. Between 300 K and 100 K, the LMCT band narrows significantly, while the optical reflectivity spectra remain unchanged below 100 K. LED irradiation of 1 at 10 and 300 K with wavelengths above 590 nm has no significant effect on the spectra, indicating that shorter wavelengths of light are required for the Mo(vi) to Mo(v) reduction.

However, irradiation at 505 nm causes a decrease in the baseline, indicating that the sample is becoming darker (Fig. 5 and S11). LED irradiation at 455 nm and below results in a clear irreversible photoinduced modification to the spectra (Fig. S11 and S12). The baseline decreases along with the emergence of a broad feature around 740 nm (seen also for 1_red_; Fig. S13), which is attributed to the presence of an intervalence charge transfer (IVCT) between Mo(v) and Mo(vi).^48,50–52^ As shown in Fig. S13, the solid-state optical reflectivity spectrum of 1_red_ clearly contrasts with the pristine spectrum of 1 and resembles the spectra observed after successive irradiations between 1050 and 365 nm (Fig. S12). Modifications to the spectra are cumulative and irreversible and heating the sample up to 350 K does not reverse the reduction process. Heating the sample above 380 K results in an irreversible modification to the spectra, which we tentatively attribute to a loss of H_2_O or THF solvent molecules from the second coordination sphere, this solvent loss is consistent with the thermo-gravimetric analysis (TGA), see Fig. S14.

### Mechanistic insights

**Fig. 6:**
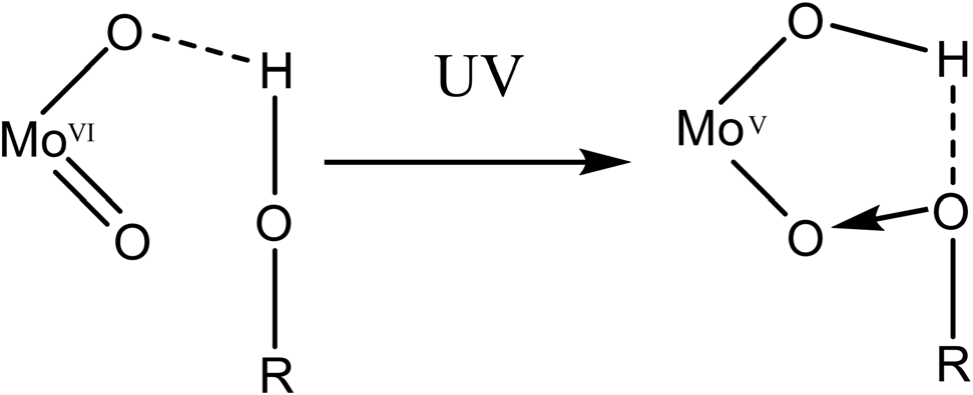
A schematic representation of the formation of the charge-transfer complex upon irradiation with UV light.

Several different mechanisms for the reduction of polyoxometalates have been studied previously.^21,51–54^ Notably, a mechanism for similar photo-induced reductions with UV light has been reported for polyoxometalate compounds.^21,50–52^ The photoexcitation of a terminal M=O bond induces a ligand-to-metal charge transfer from the terminal oxygen to the molybdenum centre. This is accompanied by a proton migration from a nearby molecule, either an alcohol (Fig. 6), water molecule or a protonated amine.^21,51,52^ The subsequent hole left in the terminal O atom is stabilised by a lone pair of electrons from the nearby O/N donor, resulting in the formation of a charge-transfer complex. In compound 1, we propose a similar photoexcitation of a terminal Mo(vi)=O bond with UV light, resulting in a LMCT. This is accompanied by an intermolecular proton transfer from the hydrogen bonded H_2_O to a bridging O atom of the POM. The hole left in the photoexcited terminal oxygen atom of the POM then interacts with a lone electron pair from the hydrogen bonded water molecule, resulting in a charge-transfer complex. However, this mechanism holds true for the reduction of only one Mo(vi) centre, due to the presence of only one hydrogen atom in close enough proximity to a bridging O atom of the polyoxometalate. Any further reduction (*vide infra*) may occur from the concurrent oxidation of ligands within the second coordination sphere and implies the existence of multiple mechanisms for the reduction.

### Magnetism

The magnetic properties of 1 and 1_Red_ constrained in eicosane were measured in an applied dc field of 1000 Oe from 280–2 K (Fig. S15). Upon cooling *χ*_M_*T* decreases steadily from 13.8 and 13.9 cm^3^ K mol^−1^ to 11.9 and 12.3 cm^3^ K mol^−1^ at 10 K for 1 and 1_Red_, respectively. This precedes a sharp drop to 5.68 (for 1) and 5.63 (for 1_red_) cm^3^ K mol^−1^ at 2 K. At 280 K, *χ*_M_*T* for 1 is slightly lower than the expected value of 14.17 cm^3^ K mol^−1^ for a free Dy(iii) ion, which can be attributed to ligand field effects.^55^ The 280 K *χ*_M_*T* value for 1_Red_ is also lower than the calculated value of 14.305 cm^3^ K mol^−1^ for a free Dy(iii) ion (14.17 cm^3^ K mol^−1^) and ‘0.36’ e^−^ (*vide supra*) from Mo(v) (0.135 cm^3^ K mol^−1^, assuming *g*_Mo_ = 2). Magnetisation *vs.* field measurements were conducted on 1 and 1_Red_ at 2, 4 and 6 K from 0–7 T, Fig. S16. At 2 K, under an applied dc field of 7 T, experimental magnetisation values (*M*_exp_) of 5.0 and 5.1 N*µ*_B_ are observed for 1 and 1_Red_, respectively. These values are in good agreement with the expected *M*_Sat_ value (≈5 N*µ*_B_) for an anisotropic *m*_J_ = ±15/2 ground state.^56^

**Fig. 7:**
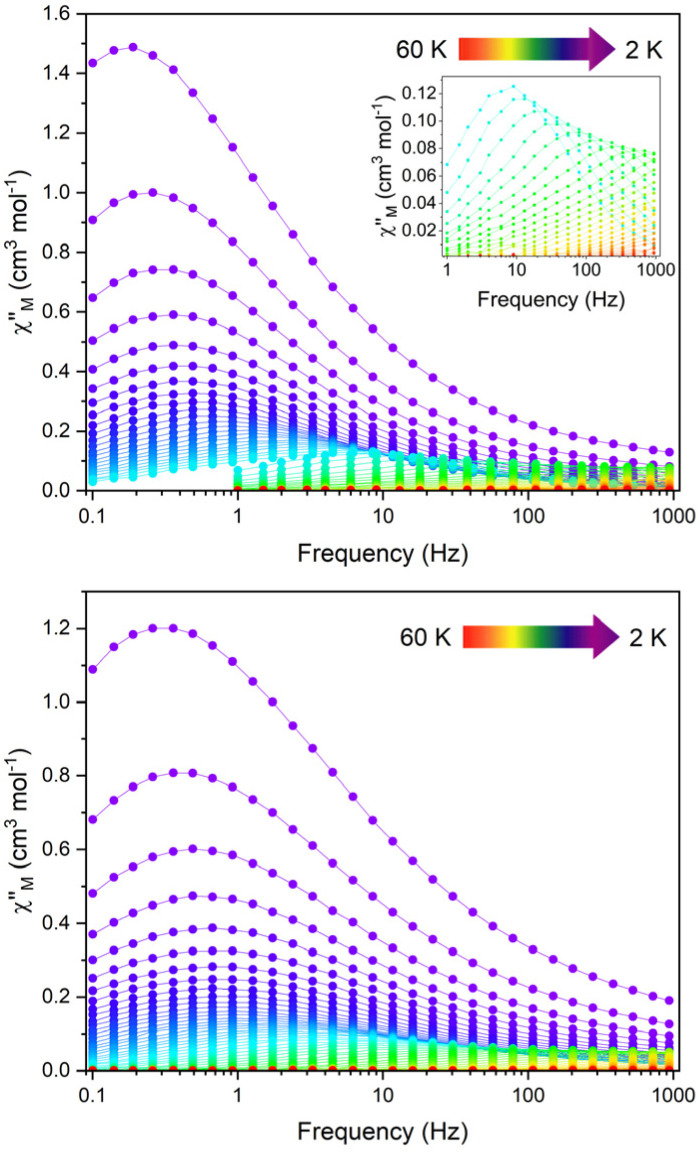
The frequency dependence of the out-of-phase 
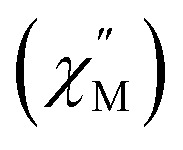
 ac susceptibility signals in the temperature range 2–60 K under zero applied dc field of 1 (top) and 1_Red_ (bottom). The out-of-phase magnetic susceptibility of 1 from 26–60 K (inset).

**Fig. 8:**
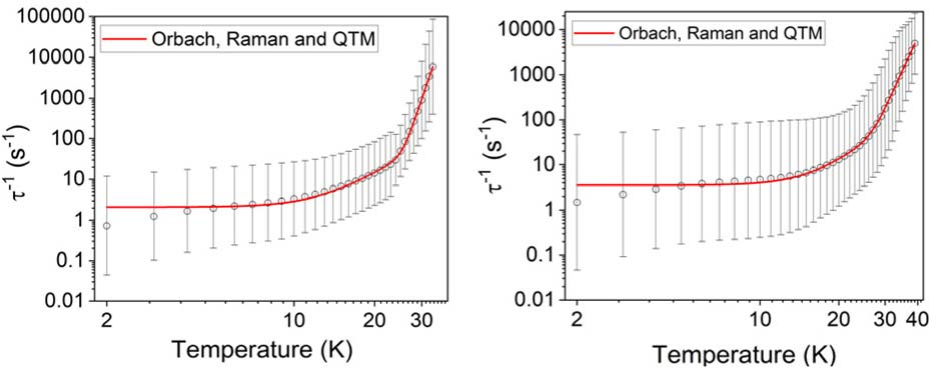
Temperature dependence of 1/*τ* for 1 (left) and 1_Red_ (right) in zero dc field. The red line represents the best fit to Orbach, Raman and QTM relaxations. Black vertical bars are estimated standard deviations in the relaxation times derived from Debye fits according to ref. 57.

Variable-temperature ac susceptibility measurements for 1 and 1_Red_ were conducted from 2–60 K with an AC frequency between 0.1 and 941 Hz under zero applied field (Fig. 7, S17 and S18). Frequency dependent out-of-phase susceptibility, *χ*″, peaks are observed up to 33 and 38 K for 1 and 1_Red_, respectively. Below 10 K, the *χ*″ peaks lose their frequency dependence, indicating the onset of efficient QTM at low temperatures in both compounds. Cole–Cole plots were then constructed from the *χ*′ *vs. χ*″ data for 1 and 1_Red_ (Fig. S19 and S20). Relaxation times, *τ*, were obtained from fitting each temperature within the Cole–Cole plot to a generalised Debye law using CCFIT2 across a 2–33 K and 2–38 K data range for 1 and 1_Red_, respectively.^57,58^ Plotting of the relaxation rate (*τ*^−1^) *vs.* temperature (Fig. 8) allows for the relaxation rate to be modelled and the extraction of the relaxation parameters using Orbach, Raman and QTM relaxation mechanisms and eqn (1).1
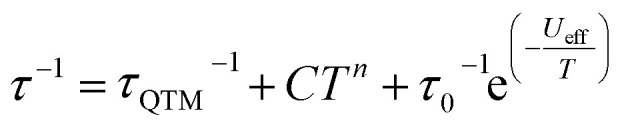


**Fig. 9:**
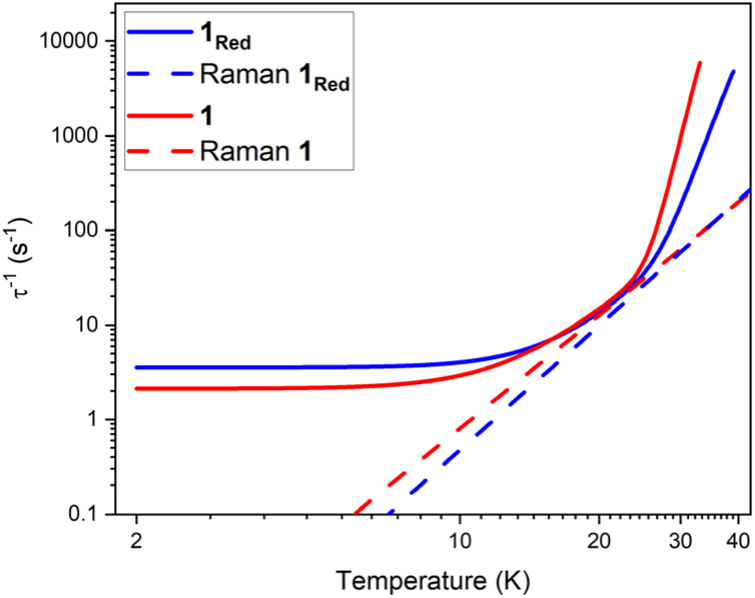
A comparison of the fits of the relaxation rates in compounds 1 (red) and 1_Red_ (blue) under zero field and the respective Raman relaxation contributions as red and blue dashed lines.

The parameters *U*_eff_ = 618(5) K, *τ*_0_ = 1.26(17) × 10^−12^ s, *C* = 8.6(5) × 10^−5^ K^−*n*^ s^−1^, *n* = 3.97(17) and *τ*_QTM_^−1^ = 2.11(28) s^−1^ were obtained for 1 across a 2–33 K range. For 1_red_, the parameters *U*_eff_ = 473(5) K, *τ*_0_ = 1.18(15) × 10^−9^ s, *C* = 2(1) × 10^−5^ K^−*n*^ s^−1^, *n* = 4.39(23) and *τ*_QTM_^−1^ = 3.56(24) s^−1^ were obtained from the fit across a 2–38 K temperature range. Compound 1 displays a large *U*_eff_ energy barrier (618(5) K), which is higher than that of the triflate precursor P1 (562(7) K). *τ*_0_ is similar to those obtained across the [Dy(Cy_3_PO)_2_(H_2_O)_5_]^3+^ family and falls within the range expected for a high-performance Dy(iii) SMM.^5,32,59^ The large *U*_eff_ in 1 is attributed to a significantly large intermolecular Dy⋯POM distance of 10.7 Å and the {Dy(H_2_O)_5_} plane–POM anion angle of 27.5°. A distancing of the negative charge of the POM further away from the Dy(iii) centre and out of the equatorial plane is expected to reduce the transverse crystal field parameters and increase the *U*_eff_ barrier in 1.^31^ The reduction of the polyoxometalate leads to a significant decrease in *U*_eff_ from 618(5) K to 473(5) K in 1_Red_. This decrease in *U*_eff_ may be related to the proton transfer from an equatorial co-crystallised H_2_O solvent molecule upon formation of a charge transfer complex (Fig. S21). In the proposed mechanism, the proton transfer may increase the equatorial electron density around the Dy(iii) ion and lead to a reduction of the axial crystal field splitting.^31^ However, the change in *U*_eff_ should not be attributed to a to a single effect and it is likely influenced by small changes in hydrogen bonding leading to varied vibrational, electronic and steric effects. Attempted deconvolution of the extent of these contributions would be unwise. However, despite the lower *U*_eff_, 1_Red_ possesses out-of-phase ac magnetic susceptibility peaks up to 38 K compared to only 33 K in 1, which suggests that 1_Red_ may exhibit magnetic blocking at higher temperatures (*vide infra*). Subtle changes to the primary or secondary coordination sphere can induce a change in the available vibrational modes within a crystal lattice causing a shift in the temperature dependence of the Raman relaxation regime.^31,60^ As expected, due to their isostructural nature, the Raman exponent (*n*) is similar across both compounds (*n* = 4.39(23) *vs.* 3.97(17) for 1 and 1_Red_, respectively), see Fig. 9. However, a fourfold decrease in the Raman coefficient *C* in 1_Red_ (2(1) × 10^−5^*vs.* 8.6(5) × 10^−5^ K^−*n*^ s^−1^ in 1) indicates a suppression of Raman relaxation and a shift in the temperature dependence of the rate of Raman relaxation to higher temperatures. This change may be attributed to a possible change in the hydrogen bonding network within the crystal lattice (Fig. S21).^35^

A small increase in *τ*_QTM_^−1^ between 1 and 1_Red_ (2.11(28) s^−1^*vs.* 3.56(24) s^−1^) may also be attributed to the changes in the second coordination sphere caused by the photoreduction of the polyoxometalate. The increased charge of the polyoxometalate and increased electron density around the equatorial region of the Dy(iii) centre are expected to induce transverse crystal field terms that increase the rate of QTM. However, this small change in *τ*_QTM_^−1^ is well within the error bars associated with the low temperature relaxation times. This highlights the need to explore compounds that have undergone further reduction in order to draw reliable conclusions about the effects on the quantum tunnelling regime (*vide infra*).

The field cooled (FC) and zero-field cooled (ZFC) magnetic susceptibilities of 1 and 1_Red_ were measured from 2–30 K, with a divergence in the field cooled and zero-field cooled magnetic susceptibilities at 8 K and 10 K, respectively (Fig. S22). The increased temperature at which 1_Red_ shows a divergence of the FC and ZFC magnetic susceptibility is perhaps surprising given the decrease in *U*_eff_, although there are some limitations in using *U*_eff_ as a guideline for designing complexes that exhibit magnetic bistability.^59^ Numerous examples of large *U*_eff_ barriers not translating to open hysteresis loops are known.^4,31,32,61^ This has been regularly attributed to prevalent through-barrier mechanisms such as Raman and QTM.^62,63^ Thus, we attribute the increased magnetic blocking in 1_Red_ to the fourfold decrease of the Raman *C* coefficient. This suppression of Raman relaxation, which manifests at low temperatures, results in an increase in the magnetic blocking. Yu *et al.* showed that the functionalisation of axial ligands can contribute to both suppression of Raman relaxation and QTM and a decrease in *U*_eff_, where the complex with a smaller *U*_eff_ displayed a five-fold increase in *T*_B(Hyst)_.^63^

**Fig. 10:**
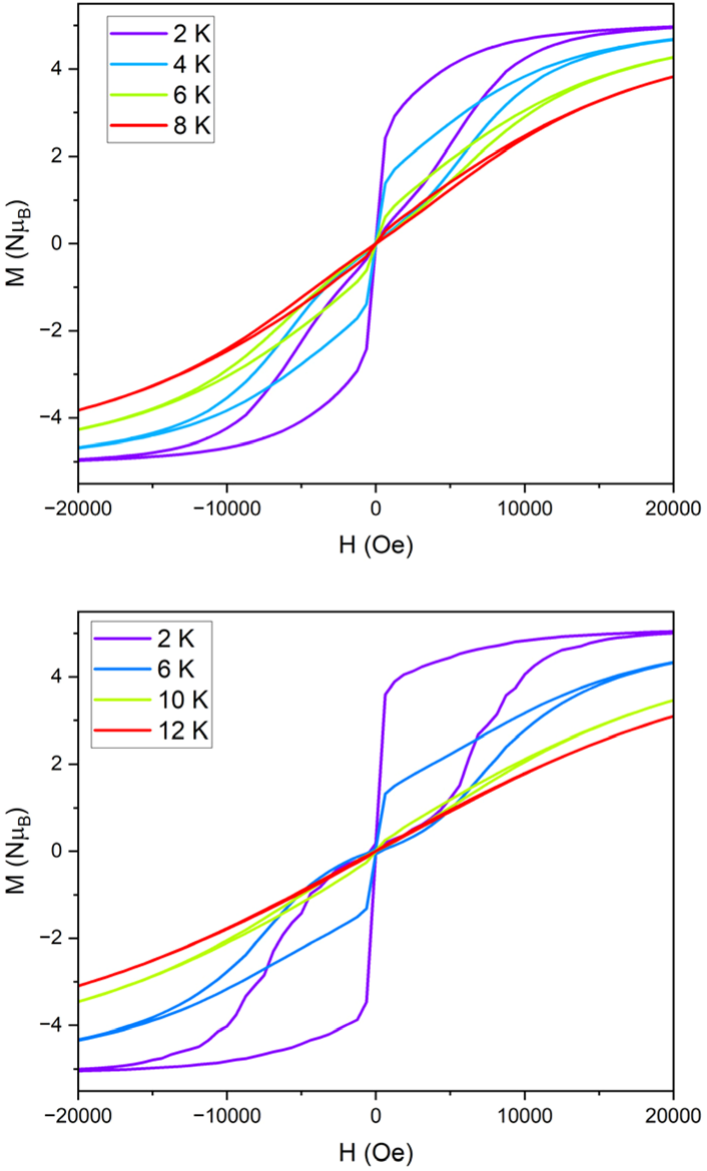
The M *vs.* H hysteresis measurements for 1 (top) and 1_Red_ (bottom) conducted with a sweep rate of 25 Oe s^−1^.

Field dependence of the magnetisation data were measured with sweep rates of 25 Oe s^−1^ for compounds 1 and 1_Red_, displaying open, waist-restricted hysteresis loops with a *T*_B(Hyst)_ of 8 and 10 K, respectively (Fig. 10). Increasing the sweep rate to 200 Oe s^−1^ results in *T*_B(Hyst)_ of 12 and 18 K for 1 and 1_Red_, respectively (Fig. S23 and S24). Despite the slightly faster rate of QTM in 1_Red_, hysteresis loops remain open at higher temperatures than in 1. The increased *T*_B(Hyst)_ in 1_Red_ is also attributed to a suppression of Raman relaxation at lower temperatures as suggested above. This can be characterised by the difference in the shapes of the hysteresis loops. Both compounds display a rapid demagnetisation close to zero magnetic field which is caused by the quantum tunnelling of the magnetisation. However, the narrower shape of the hysteresis loops in 1 indicates a faster loss of magnetisation at non-zero magnetic fields. We attribute this to the faster rate of Raman relaxation, obtained from our analysis of the ac susceptibility data. The underlying mechanism for the enhancement of *T*_B(hyst)_ and *T*_B(ZFC)_ likely stems from a combination of vibrational and electronic effects resulting from the formation of a charge transfer complex (Fig. S21). The formation of a charge transfer complex is expected to increase the partial charge of the H_2_O molecule that is hydrogen bonded to both the POM and the [Dy(H_2_O)_5_(Cy_3_PO)_2_]^3+^ molecule. This is expected to generate stronger hydrogen bonding between the second coordination sphere of the equatorial H_2_O molecules of [Dy(H_2_O)_5_(Cy_3_PO)_2_]^3+^ and increase the partial charge of the equatorial H_2_O molecule. As a result, we should expect an increased electron density in the equatorial region of [Dy(H_2_O)_5_(Cy_3_PO)_2_]^3+^ and a decrease in *U*_eff_. Conversely, the increased strength of the hydrogen bonding is expected to correspond to weaker out of plane H_2_O bending modes. A shift of these vibrations to a higher frequency may explain the suppression of Raman relaxation in 1_Red_ and an increase in *T*_B(hyst)_ and *T*_B(ZFC)_.

### Optical dilution of 1_Red_

**Fig. 11:**
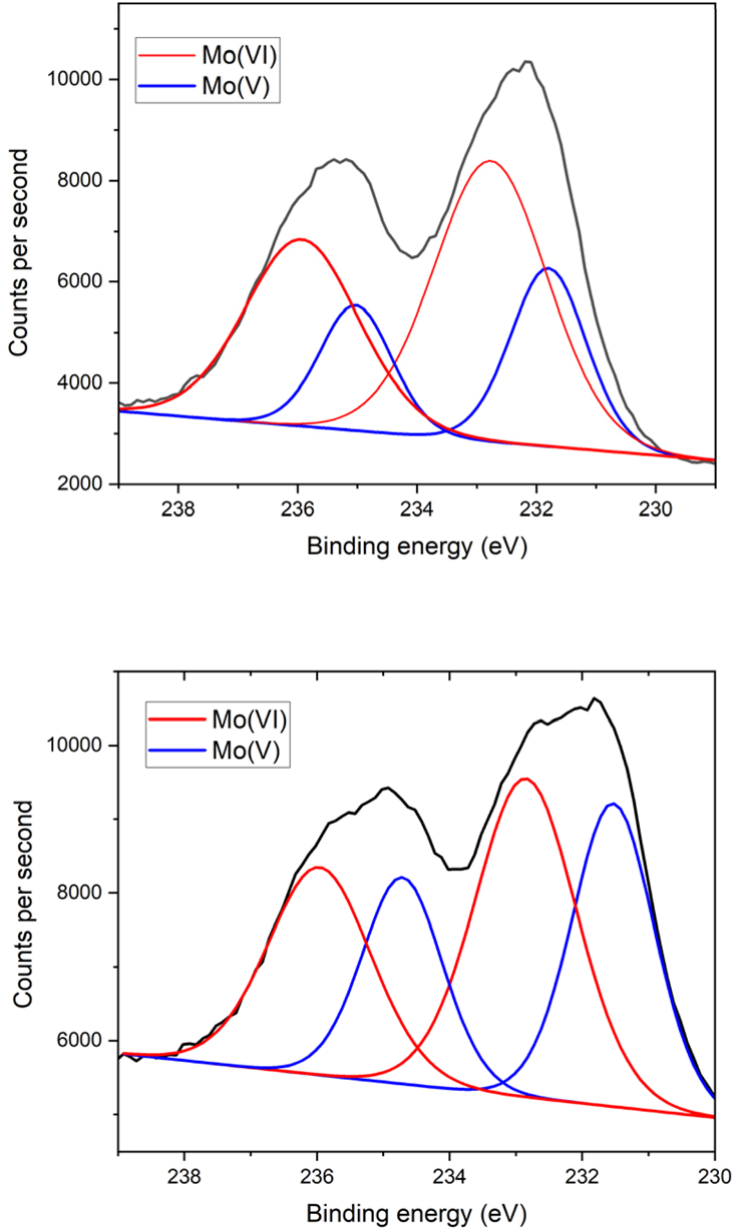
The initial Mo 3d XPS spectra of compound 1_Red_@KBr (top) and 
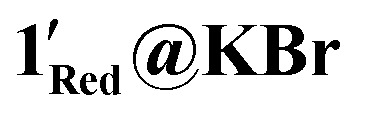
 (bottom) where the black line represents the counts per second, the red line represents the fit to Mo(vi) and the blue line is the fit to Mo(v).

Following our observation of a light-induced reduction of 1, grinding in an optically dilute diamagnetic compound (KBr) was conducted to decrease the optical density and enhance the reduction of Mo(vi). Two samples were prepared: 1_Red_@KBr (with a mass ratio of 1 : 9.45 1_Red_ : KBr, prepared from 5.31 mg of 1_Red_ and 50.18 mg KBr) and 
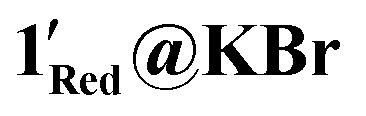
 (with a mass ratio of 1 : 29.4 1_Red_ : KBr, prepared from 2.65 mg of 1_Red_ and 77.87 mg of KBr) and each was ground for 30 minutes. Subsequently, these samples were compressed into translucent discs and kept in indirect sunlight on a benchtop for 2 weeks. XPS measurements were conducted on 1_Red_@KBr and 
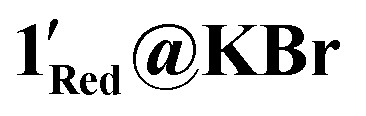
 and two Mo(vi) 3d signals are observed at binding energies of approximately 233 and 236 eV, as seen in 1_Red_. However, the Mo(v) signals at ∼232 and 235 eV are much larger than those observed in 1_Red_ (Fig. 11). Decomposition of the 3d doublets in 1_Red_@KBr and 
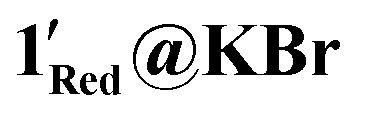
 results in 1 : 2.4 and 1 : 1.3 ratios of Mo(v) to Mo(vi), respectively. This equates to 29.8 and 43.7% Mo(v) or ‘3.58’ and ‘5.24’ Mo(v) centres per POM in 1_Red_@KBr and 
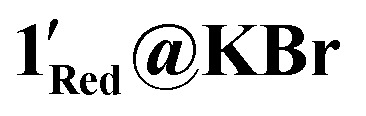
, respectively. This significant increase in Mo(v)% is attributed to the reduced optical density of the samples, allowing UV-light to photo-reduce an increased number of POM centres. However, the presence of over 8% Mo(v) strongly indicates a second mechanism (*vide supra*) for the reduction of the Mo(vi) centres. Only one H_2_O molecule is hydrogen bonded to a bridging O atom on the POM, indicating that only one Mo(vi) centre should be reduced by this mechanism. A second potential mechanism is the concurrent oxidation of molecules within the second coordination sphere, such as diethyl ether and THF, which can be commonly oxidised.^64,65^

The magnetic susceptibility of 1_Red_@KBr was measured under an applied dc field of 1000 Oe from 290–2 K (Fig. S26). The *χ*_M_*T* product at 290 K in 1_Red_@KBr (15.7 cm^3^ K mol^−1^) is much larger than the expected room temperature *χ*_M_*T* value for a single Dy(iii) ion (14.17 cm^3^ mol^−1^ K). This large increase can be attributed to the presence of ‘3.58’ Mo(v) centres in 1_Red_@KBr, which would be expected to yield a room temperature *χ*_M_*T* value of 15.5 cm^3^ K mol^−1^, assuming *g*_Mo_ = 2.

**Fig. 12:**
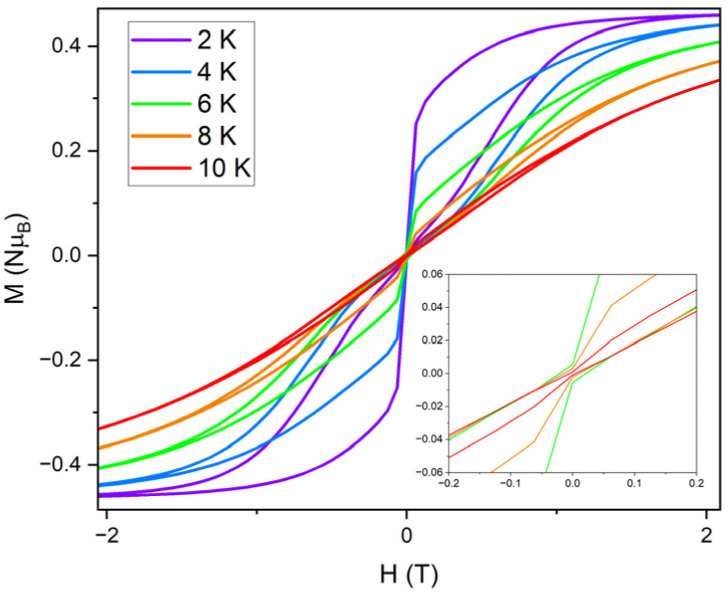
M *vs.* H hysteresis measurements of 1_Red_@KBr conducted from 2–10 K, with a sweep rate of 25 Oe s^−1^, zoomed in around zero field (inset).

**Fig. 13:**
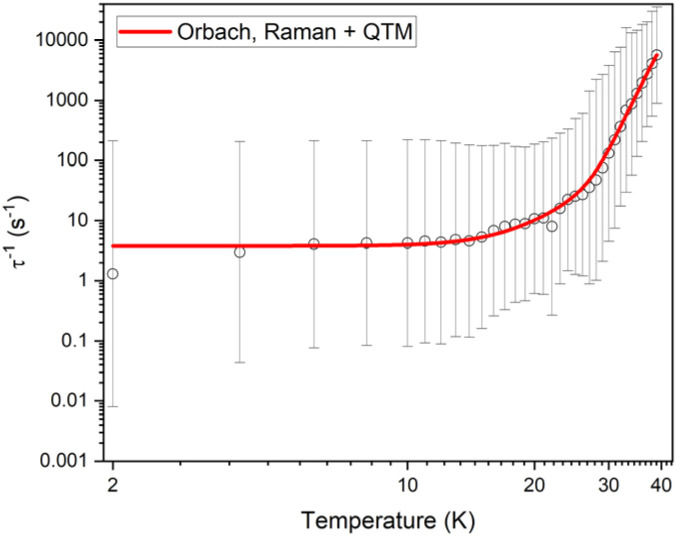
Temperature dependence of 1/*τ* for 1_Red_@KBr in zero dc field. The red line represents the best fit to Orbach, Raman and QTM relaxation. Black vertical bars are estimated standard deviations in the relaxation times derived from Debye fits according to ref. 57.

The FC and ZFC magnetic susceptibilities were measured from 2–30 K, with a divergence in the FC and ZFC magnetic susceptibilities at 10 K for 1_Red_@KBr (Fig. S27). The retention of magnetic blocking in a highly reduced system highlights the robust nature of the [Dy(H_2_O)_5_(Cy_3_PO)_2_]^3+^ complex and its potential to exhibit magnetic bistability upon significant changes to the second coordination sphere. Field dependence of the magnetisation data were measured on 1_Red_@KBr at a sweep rate of 25 Oe s^−1^ (Fig. 12), revealing that the compound maintains open hysteresis loops at 2–10 K, again despite the significant reduction of the polyoxometalate. The persistence of hysteresis loops up to the same temperatures as 1_Red_ indicates that the Dy(iii) complex in 1_Red_@KBr is able to act as a high-performance SMM, despite the reduced environment. To further investigate the viability of 1_Red_@KBr as a functioning SMM-hybrid material, variable-temperature ac susceptibility measurements for 1_Red_@KBr were conducted from 2–60 K for ac frequency between 0.1 and 941 Hz under zero applied field (Fig. S28). Frequency dependent *χ*″ susceptibility peaks are observed up to 38 K. Below 10 K, the *χ*″ peaks lose their frequency dependence, indicating the onset of efficient QTM at low temperatures, as seen in 1 and 1_Red_. Relaxation times were determined from Cole–Cole plots of *χ*′ *vs. χ*″ data from 2–38 K using a generalised Debye model, see Fig. S29.^57,58^ Plotting the relaxation rate (*τ*^−1^) *vs.* temperature allows for the relaxation rate to be modelled and the extraction of the relaxation parameters using Orbach, Raman and QTM relaxation mechanisms and eqn (1) (Fig. 13). The parameters *U*_eff_ = 520(9) K, *τ*_0_ = 2.9(7) × 10^−10^ s, *C* = 1.5(5) × 10^−6^ K^−*n*^ s^−1^, *n* = 5.1(6) and *τ*_QTM_^−1^ = 3.8(6) s^−1^ were obtained from the fit across a 2–38 K temperature range. A comparison of the parameters and fits obtained for 1, 1_Red_ and 1_Red_@KBr can be found in Table 1 and Fig. S30. Whilst the increased reduction of the polyoxometalate has little impact on *T*_B(Hyst)_ and *T*_IRREV_, a noticeable change in the Orbach and Raman relaxation is observed. An order of magnitude decrease in the Raman *C* coefficient compared to 1_Red_ may be related to the increased reduction of the polyoxometalate. The addition of further electron density to the polyoxometalate is expected to increase the length of the Mo–O bonds. As such we can expect this to result in a shift of the available vibrational modes (phonon density of states), which may explain the significant change in Raman relaxation.^63^ However, diluting the sample into KBr may have a more significant contribution towards the change in Raman relaxation. KBr is an inorganic solid and is expected to possess vastly different vibrational modes compared to molecular crystals.^66^

**Table 1:** A comparison of *T*_B(Hyst)_, *T*_IRREV_, *U*_eff_, *τ*_0_, *C*, *n* and *τ*_QTM_^−1^ parameters for 1, 1_Red_, and 1_Red_@KBr

Compound	1	1_Red_	1_Red_@KBr
*T*_B(Hyst)_ a (K)	8	10	10
*T*_IRREV_ (K)	8	10	10
*U*_eff_ (K)	618(5)	473(5)	520(9)
*τ*_0_ (s)	1.3(2) × 10^−12^	1.2(2) × 10^−9^	2.9(7) × 10^−10^
*C* (K^−*n*^ s^−1^)	8.6(5) × 10^−5^	2(1) × 10^−5^	1.5(5) × 10^−6^
*n*	3.97(17)	4.4(2)	5.1(6)
*τ*_QTM_^−1^ (s^−1^)	2.1(3)	3.6(2)	3.8(6)

a Sweep rate of 25 Oe s^−1^

The increased *U*_eff_ compared to 1_Red_ is not easily explained by considering the reduction of the polyoxometalate alone, especially considering the decreased *U*_eff_ upon the reduction of 1 to 1_Red_. Instead, we suggest that changes in the Raman relaxation may, to some extent, overlap with the Orbach relaxation and result in a fit where it is difficult to differentiate between the two mechanisms. Most remarkable is the negligible change in *τ*_QTM_^−1^ between 1_Red_ and 1_Red_@KBr despite the dramatic increase in Mo(v) content 3% *vs.* 29.8%, respectively. The increased reduction of the polyoxometalate, which lies in an equatorially offset region from the Dy(iii) complex is expected to increase the rate of quantum tunnelling by increasing transverse crystal field terms. However, in 1_Red_@KBr we observe no substantial increase in *τ*_QTM_^−1^ compared to 1_Red_. This correlates well with the persistence of *T*_B(Hyst)_ up to 10 K in both 1_Red_ and 1_Red_@KBr, where the waist restriction and ultimate closing of these loops is governed by the rate of quantum tunnelling of the magnetisation. We propose that the persistence of SMM behaviour in 1_Red_@KBr is due to the appreciable distance between the polyoxometalate and the SMM and the robust nature of the pentagonal bipyramidal [Dy(H_2_O)_5_(Cy_3_PO)_2_]^3+^ SMM.

## Conclusions and future work

We have designed a high-yield, air-stable anion exchange synthesis to achieve high performance polyoxometalate–Dy(iii) SMM hybrid compounds, which display photoreduction capabilities. The observation that the pentagonal bipyramidal [Dy(H_2_O)_5_(Cy_3_PO)_2_]^3+^ SMM still possesses impressive magnetic behaviour despite the presence of a significant POM charge nearby, may have important applications when attempting to integrate single-molecule magnets into devices. Using KBr dilution to enhance the photo-reduction of 1_Red_ presents a new method for investigating the magnetic properties of photoactive SMM materials. It also presents a quantitative method to investigate the effect of Mo(v)% on the magnetic properties of 1_Red_, a study which is currently underway. It also opens up opportunities to investigate the effect the KBr diamagnetic matrix has on vibrational relaxation mechanisms such as Raman and Orbach relaxation, alongside the investigation of other potential matrices. Future studies could benefit from the inclusion of bulk-sensitive techniques such as XAS to characterise further the redox behaviour. Looking ahead, the resistance of [Dy(H_2_O)_5_(Cy_3_PO)_2_]^3+^ to nearby charge and the conductive capabilities of polyoxometalates may present the opportunity to study the effect of electrical currents on the performance of these materials. Furthermore, the highly reduced nature of the polyoxometalate in 1_Red_@KBr may also present an interesting energy landscape, giving rise to a conduction band and a route into multi-functional materials, further advancing the integration of SMMs into new devices.

## Experimental methods

### Synthesis and characterisation

All reagents were used as received without further purification. No safety hazards were encountered during the described experimental procedures.

#### Synthesis of [Dy(H_2_O)_5_(Cy_3_PO)_2_](CF_3_SO_3_)_3_·2(Cy_3_PO) (P1)

Dy(CF_3_SO_3_)_3_ (0.6 mmol, 378 mg) and Cy_3_PO (2.4 mmol, 710 mg) were dissolved in 20 mL ethanol and heated at 70 °C for 5 hours. The resulting solution was filtered and layered with Et_2_O at room temperature giving colourless crystals within hours, with a yield of 60%. Elemental analysis calculated % for C_75_H_142_DyF_9_O_18_P_4_S_3_: C, 47.77%; H, 7.59%; N, 0%. Found: C, 47.40%; H, 7.49%; N, 0%.

#### Synthesis of [Dy(H_2_O)_5_(Cy_3_PO)_2_][Mo_12_PO_40_]·2(Cy_3_PO)·4THF·2H_2_O·Et_2_O (1)

(P1) (0.1 mmol, 189 mg) was dissolved in 5 mL of hot THF and H_3_[Mo_12_PO_40_]·24H_2_O (0.1 mmol, 226 mg) was dissolved in 5 drops of deionised water. The solutions were combined producing a yellow precipitate and the mixture was heated at 60 °C for 1 hour. The precipitate was removed by filtration and yellow block crystals suitable for single crystal X-ray diffraction were obtained after 1–2 days, by slowly diffusing cold Et_2_O into the solution. Yield ∼70%. Elemental analysis calculated % for C_88_H_180_DyMo_12_O_55_P_5_ (–Et_2_O): C, 29.47%; H, 5.06%; N: 0%. Found: C, 29.38%; H, 4.82%; N: 0%. Selected IR data: *v̄* (cm^−1^) 794, 872, 952, 1060, 1446, 1648, 2342, 2362, 2853, 2930.

## Author contributions

EL carried out the synthesis and sample characterisation. EL and ABC carried out the magnetic measurements and data analysis, advised by MM. EL and SD carried out the crystallographic measurements and data analysis, advised by CW. CK carried out the XPS measurements and data analysis. MR and RC carried out the optical measurements and data analysis. MM and ABC devised the initial concept of using a POM anion and MM supervised the project. The manuscript was written by EL and MM with input from all authors.

## Conflicts of interest

There are no conflicts to declare.

## Supplementary Material

SC-017-D5SC07950K-s001

SC-017-D5SC07950K-s002

## Data Availability

The data supporting this article have been included as part of the supplementary information (SI). Supplementary information: further experimental details and supporting data. See DOI: https://doi.org/10.1039/d5sc07950k. CCDC 2469864 (1) and 2495217 (1_Red_) contain the supplementary crystallographic data for this paper.^67*a*,*b*^
