# Supplementary material for: Highly reducible polyoxometalate–Dy(iii) SMM hybrid materials with exceptional charge stability
Source: Chem Sci. 2025 Nov 18;17(6):3248–58. doi: 10.1039/d5sc07950k (PMC12709713; doi:10.1039/d5sc07950k)
Supplement: SC-017-D5SC07950K-s001 [file SC-017-D5SC07950K-s001.pdf]

Supplementary Information (SI) for Chemical Science.
This journal is © The Royal Society of Chemistry 2025

ELECTRONIC SUPPLEMENTARY INFORMATION

Highly reducible polyoxometalate–Dy(III) SMM hybrid materials with exceptional charge stability

Ethan Lowe,[a] Mathieu Rouzières,[b] Sarah K. Dugmore, [a] Christopher Kelly,[a] Claire Wilson,[a] Angelos B. Canaj,[ac] Rodolphe Clérac*[b] and Mark Murrie*[a]

a. School of Chemistry, University of Glasgow, University Avenue, Glasgow, G12 8QQ, UK.
E-mail: mark.murrie@glasgow.ac.uk

b. Univ. Bordeaux, CNRS, CRPP, UMR 5031, 33600 Pessac, France.
E-mail rodolphe.clerac@u-bordeaux.fr

c. The Henry Royce Institute, Royce Hub Building, The University of Manchester, Manchester, M13 9PL.

Experimental Methods

All experiments were carried under aerobic conditions using materials and solvents as received without further purification.

Characterisation

Elemental analyses (C, H, N) were performed by the University of Glasgow microanalysis service. Powder X-ray diffraction data were collected on samples of **1**, **1**$_{\mathbf{Red}}$ and **1**$_{\mathbf{Red}}$**@KBr** on a Rigaku MiniFlex benchtop diffractometer equipped with a Cu sealed tube X-ray source (λ (CuKα) = 1.5405 Å) and a 6-position sample changer on zero-background silicon sample holders at the University of Glasgow.

Crystal Structures

Single crystal X-ray diffraction data for **1** and **1**$_{\mathbf{Red}}$ were collected at 100 K on a Bruker D8 VENTURE diffractometer equipped with a Photon II CPAD detector, dual (Cu and Mo) ImS 3.0 microfocus sources, and an Oxford Cryosystems Cryostream 1000 device. The data sets were collected for **1** using Mo-Kα radiation (λ = 0.71073 Å) and for **1**$_{\mathbf{Red}}$ using Cu-Kα radiation (*λ* = 1.54178 Å) and the APEX3 software package.

The structure of **1** (yellow crystals) was collected from a comparatively quick measurement, where the single crystal had not turned blue by the end of the measurement. There was a slight darkening, but indexing and creating a refinement model from the first ~200 and last ~200 frames showed no significant difference. Single crystals of **1**$_{\mathbf{Red}}$ (blue crystals) were obtained by leaving yellow single crystals of **1** in indirect sunlight for one week.

The crystals of the reduced form (**1**$_{\mathbf{Red}}$) show some deterioration in quality and consequently the diffraction data, while satisfactory are not of the same quality as for **1**. Considerable effort was made to obtain a fully anisotropic model refinement for **1**$_{\mathbf{Red}}$ but even with significant use of restraints one or two atoms didn't refine satisfactorily. We therefore chose to be consistent and keep all but the heavier atoms with isotropic adps and obtain a sensible, robust structural model.

The solvent molecules are straightforward to model in **1**, but they are less straightforward in **1**$_{\mathbf{Red}}$. Starting from the structural model of **1** and refining against the data for **1**$_{\mathbf{Red}}$ gives good overlap for the main structure, but there do appear to be differences between the solvent molecules in **1**$_{\mathbf{Red}}$ and **1**. This is consistent with the lattice solvent being involved in the reduction mechanism. A solvent mask was used for all the lattice solvent, rather than have a mixture of a poor explicit model for some solvent molecules and consequently have a less effective solvent mask. The

electron count from the solvent mask, reported in the CIF, is a good match for the solvent molecules modelled explicitly in **1**.

Magnetic characterisation

Variable-temperature, solid-state direct current (dc) magnetic susceptibility data were collected on a Quantum Design MPMS3 SQUID magnetometer at the University of Glasgow and on a Quantum Design SQUID-VSM magnetometer at the University of Edinburgh. Ac magnetic susceptibility data were collected on a Quantum Design MPMS3 SQUID magnetometer at the University of Glasgow. Microcrystalline samples were prepared using a mortar and pestle in open air and then added to gelatin capsules in the presence of eicosane or KBr. Diamagnetic corrections were applied to the observed paramagnetic susceptibilities using Pascal's constants. The diamagnetic contribution of the sample holder, eicosane and KBr were corrected by measurements.

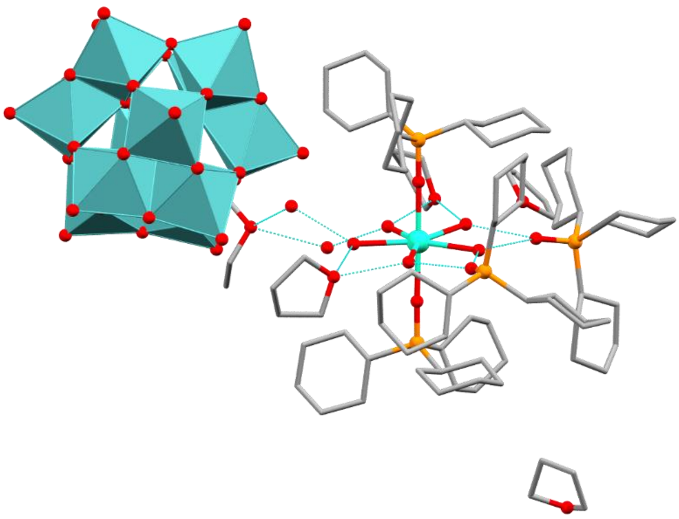

Figure S1 The relative position of the POM anion to the Dy complex in $[Dy(H_2O)_5(Cy_3PO)_2][Mo_{12}PO_{40}]\cdot 2(Cy_3PO)\cdot 4THF\cdot 2H_2O\cdot Et_2O$ (**1**) with H atoms omitted for clarity. C, grey; Dy, cyan; O, red; P, orange; Mo, light blue.

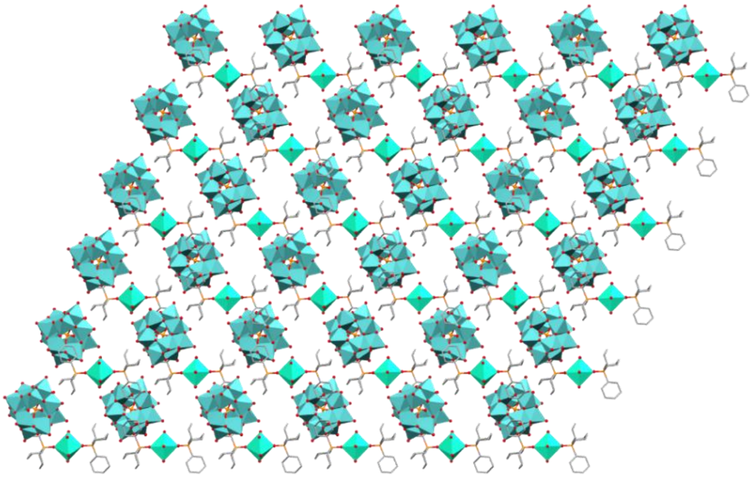

Figure S2 The crystal packing of **1** along the crystallographic *b* axis highlighting the separation between Dy(III) centres. All co-crystallised ligands and solvent molecules are omitted for clarity. C, grey; Dy, cyan; O, red; P, orange; Mo, light blue.

Table S1 Crystal Data and Structure Refinement Parameters for $[Dy(H_2O)_5(Cy_3PO)_2][Mo_{12}PO_{40}]\cdot 2(Cy_3PO)\cdot 4THF\cdot 2H_2O\cdot Et_2O$ (**1**) and the reduced compound $\mathbf{1}_{\mathbf{Red}}$.

Compound	**1**	$\mathbf{1}_{\mathbf{Red}}$
Empirical formula	$C_{92}H_{188}DyMo_{12}O_{56}P_5$	$C_{36}H_{76}DyO_7P_2\cdot Mo_{12}O_{40}P$ $\cdot 2(C_{18}H_{33}OP)$
Formula weight	3659.04	3260.48
Temperature/K	100.0	100.0
Crystal system	monoclinic	monoclinic
Space group	Cc	Cc
a/Å	24.6687(9)	24.5974(15)
b/Å	19.2311(9)	19.2115(12)
c/Å	29.3029(15)	29.5224(19)
α/°	90	90
β/°	113.233(3)	113.102(2)
γ/°	90	90
Volume /Å^3	12774.2(10)	12832.1(14)
Z	4	4
ρ_{calc} g/cm^3	1.903	1.688
μ /mm^{-1}	1.861	13.52
F(000)	7332.0	6444
Crystal size /mm^3	0.13 × 0.05 × 0.04	0.08 × 0.08 × 0.06
Radiation	MoKα (λ = 0.71073 Å)	CuKα (λ = 1.54178 Å)
2Θ range for data collection /°	2.778 to 54.256	6.036 to 158.386
Index ranges	$-31 \leq h \leq 31$, $-24 \leq k \leq 24$, $-37 \leq l \leq 37$	$-31 \leq h \leq 31$, $-24 \leq k \leq 23$, $-34 \leq l \leq 37$
Reflections collected	78217	63677
Independent reflections	27833 [R_{int} = 0.0525, R_{sigma} = 0.0575]	22126 [R_{int} = 0.0499, R_{sigma} = 0.0537]
Data/restraints/parameters	27833/2389/1543	22126/75/655
Goodness-of-fit on F^2	1.116	1.028
Final R indexes [$I \geq 2\sigma(I)$]	R_1 = 0.0691, wR_2 = 0.1423	R_1 = 0.0686, wR_2 = 0.1609
Final R indexes [all data]	R_1 = 0.0765, wR_2 = 0.1461	R_1 = 0.0694, wR_2 = 0.1617
Largest diff. peak/hole / e Å^{-3}	2.30/-1.57	1.10/-2.20
Flack parameter	0.349(19)	0.523(7)
CIF deposited with CCDC	Yes (CCDC 2469864)	Yes (CCDC 2495217)

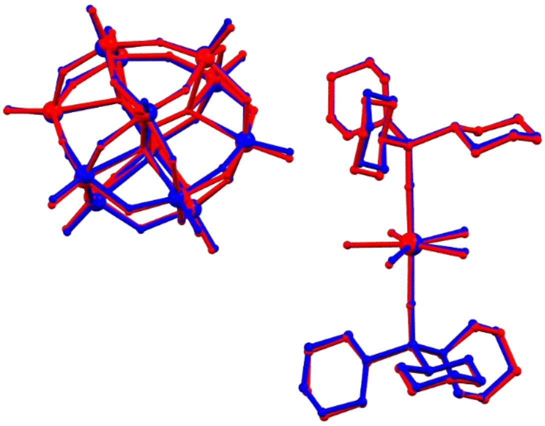

Figure S3 Overlay of the crystal structures showing the $[Dy(H_2O)_5(Cy_3PO)_2]^{3+}$ and $[Mo_{12}PO_{40}]^{3-}$ components of **1** and **1**$_{\mathbf{Red}}$.

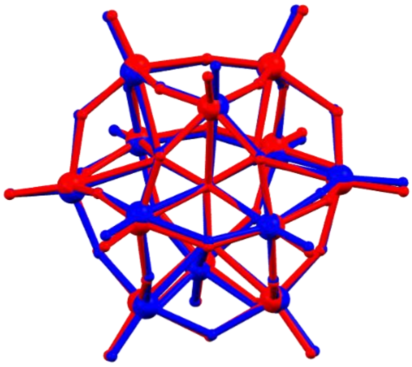

Figure S4 Overlay of the polyoxometalate components of **1** and **1**$_{\mathbf{Red}}$.

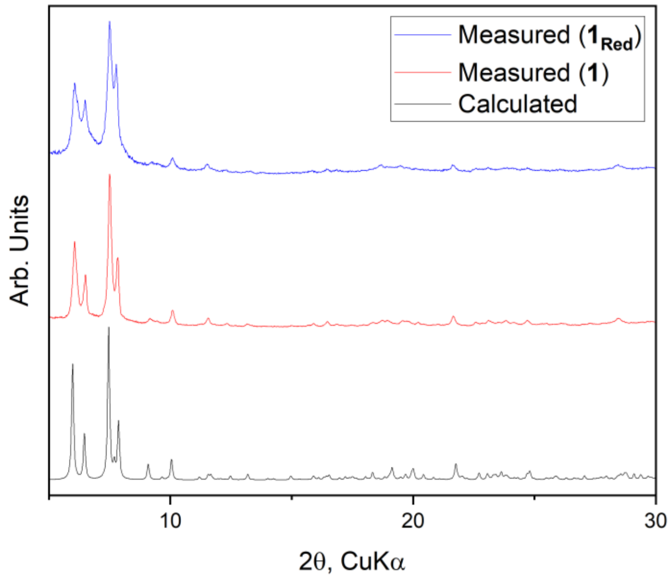

Figure S5 The powder X-ray diffraction patterns of **1** and $\mathbf{1}_{\mathbf{Red}}$, where the black line represents the calculated X-ray diffraction pattern from a single crystal of **1** collected at 100 K; the red line represents the yellow, fully ground crystalline powder sample of **1** collected at room temperature and the blue line represents the blue, fully ground crystalline powder sample of $\mathbf{1}_{\mathbf{Red}}$ collected at room temperature.

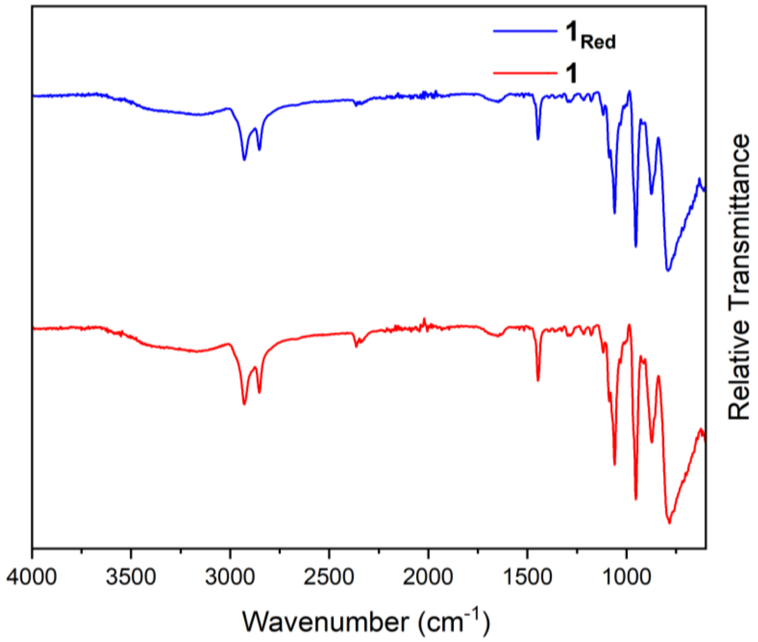

Figure S6 The IR spectra of complexes **1** and $\mathbf{1}_{\mathbf{Red}}$ at ambient temperature.

Table S2 Elemental analysis for **1** and $\mathbf{1}_{\mathbf{Red}}$, compared to the expected value for $C_{88}H_{180}DyMo_{12}O_{55}P_5$ (- Et_2O).

Element	**1** (%)	$\mathbf{1}_{\mathbf{red}}$ (%)	Expected (%)
C	29.38	29.75	29.47
H	4.82	4.84	5.06
N	0	0	0

X-ray photoelectron spectroscopy (XPS)

Experiments were conducted on a Kratos AXIS Supra+ using a monochromatic aluminium excitation source. Survey scans (as in Figure S7) were performed with an excitation current of 25 mA across a 286.690 – 1491.690 eV kinetic energy (1200 to -5 eV binding energy) range in 1 eV steps with a measurement time of 120 s. A dwell time of 100 ms was used for each survey with an X-ray power of 375.00 W. A charge neutraliser set with a filament current of 0.45A, filament bias of 1 V and charge balance of 4V, was used to remove build-up of positive charge on sample surfaces. The resultant energy shift in the spectra was compensated for by charge referencing to adventitious carbon 1s at 284.4 eV.

High-resolution scans of the Mo 3d region (approx. binding energy range of 240 - 225 eV) were performed with an excitation current of 30 mA in 0.1 eV steps with a measurement time of 60 s. A dwell time of 400 ms was used for each survey with an X-ray power of 450 W.

Individual components of the Mo 3d spectra found with the high-resolution scans were fitted with a Gaussian*Lorentzian line shape at a ratio 70% Gaussian to 30% Lorentzian. The four components were identified as the $3d_{3/2}$ electrons of Mo(V) at 231.5 eV and Mo(VI) at 233 eV, and the $3d_{1/2}$ electrons of Mo(V) at 234 eV and Mo(VI) at 236 eV. A standard d-shell spin-orbit splitting ratio of 3:2 for the 3/2 to 1/2 electrons was used as a constraint in the fitting for each of the Mo(V) and Mo(VI) doublets, with Mo 3d spin-orbit splitting Δ = 3.155 eV.

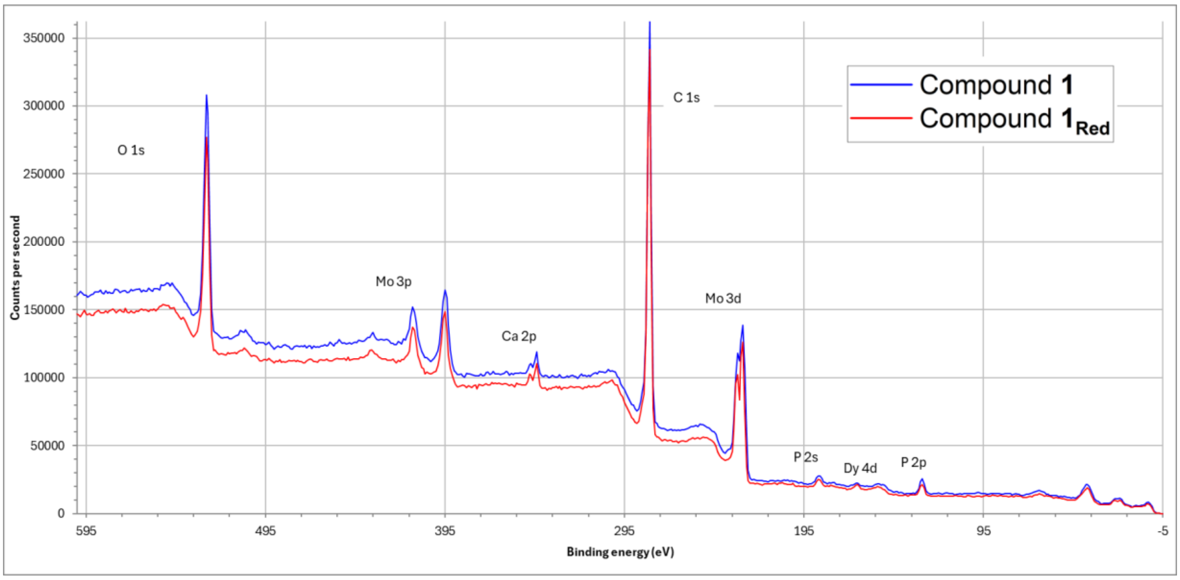

Figure S7 The wide scan XPS spectra of compound **1** (blue line) and $\mathbf{1}_{\mathbf{Red}}$ (red line).

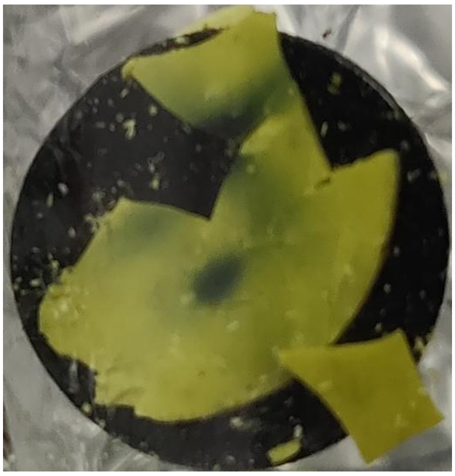

Figure S8 A photograph of **1** after XPS measurement, showing where the sample has turned blue at the point of measurement.

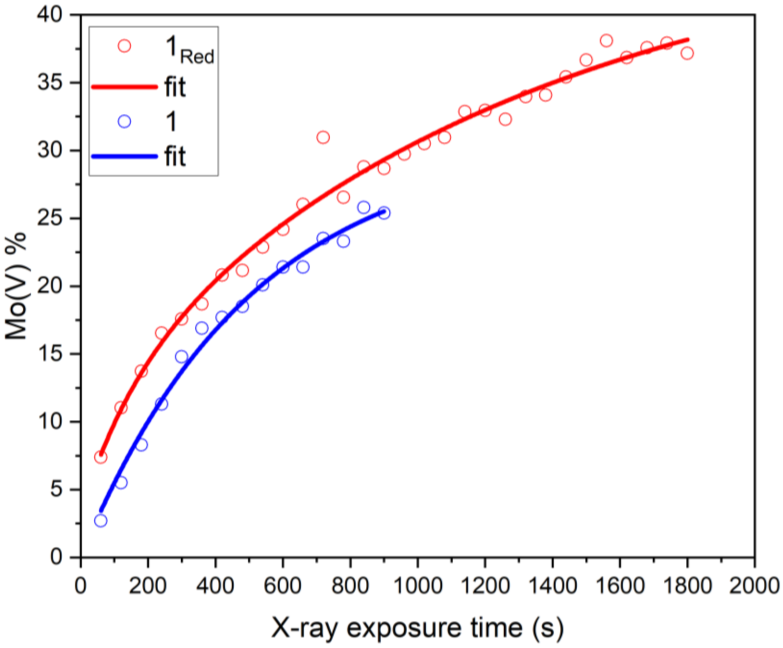

Figure S9 The Mo(V) % observed from deconvolution of the respective XPS spectra at each time interval for **1** (blue) and $\mathbf{1}_{\mathbf{Red}}$ (red). The red and blue lines represent the fit to a stretched exponential and should only be used as a guide.

Solid-state optical reflectivity

Optical reflectivity measurements have been performed with a home-built system, operating in the range of 400 to 1000 nm and installed in a commercial Quantum Design Physical Properties Measurement System device to manage a precise temperature control from 2 to 300 K. The optical probe is divided into three optical fibres, to connect to a visible CCD high-sensitivity spectrometer (Hamamatsu TM-VIS/NIR C10083CA), to a white light source (Leica CLS 150 XD tungsten halogen source adjustable from 0.05 $mW \cdot cm^{-2}$ to 1 $W \cdot cm^{-2}$), and to a homemade Light-emitting diodes (LEDs) irradiation box. This LED box is equipped with 14 high power LEDs (Thorlabs mounted high power LED series) to provide an adjustable irradiation for wavelength from 365 to 1050 nm and for power up to 16 mW/m^2. The sample is placed at the other end of the optical probe in a sample holder. The light is thus reflected on the sample surface before measurement with the spectrometer. Because of possible grain size effect on measurement, samples are carefully ground and gently pressed into the sample holder to obtain a surface as flat as possible. The measurements were calibrated with barium sulfate as the reference sample. As the samples are potentially photosensitive, the light exposure time was minimized during the experiments keeping the samples in the dark except during the measurements when white light is shined on the sample surface (P = 0.08 $mW \cdot cm^{-2}$). A full automation of the experimental setup provides some sequential operations (acquisition, irradiation and temperature control).

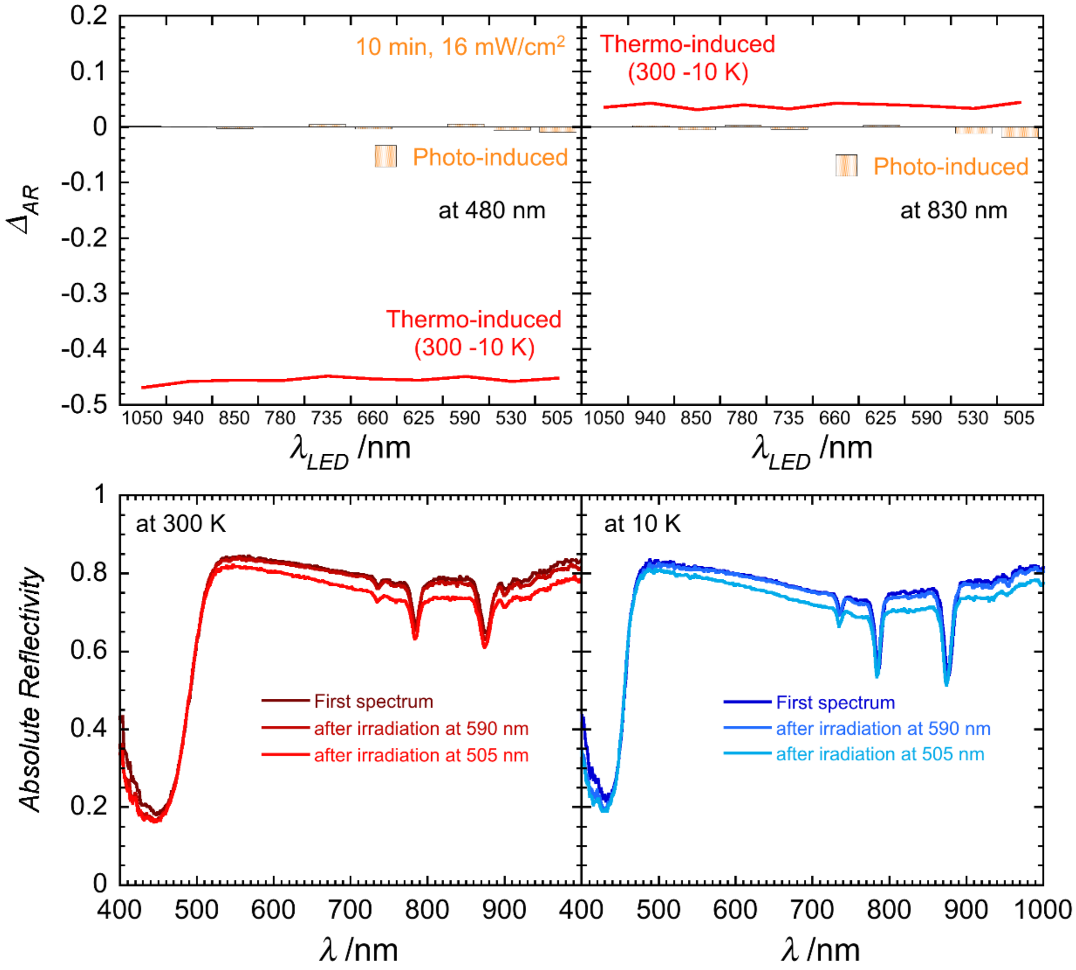

Figure S10 LED irradiations of **1** from 1050 to 505 nm. *Top:* after a fast cooling of **1** from 300 K in the dark, the plots show the variation of the absolute optical reflectivity at 10 K comparing before and after successive irradiations with different LEDs from 1050 nm to 505 nm ($\Delta_{AR} = AR_{after} - AR_{before}$; 10 minutes, at 16 mW cm^{-2}) recorded at 480 nm (left) and at 830 nm (right). Note that between each irradiation, the sample was heated to 300 K and cooled down to 10 K in the dark. For comparison, the red line is the thermal variation of the absolute optical reflectivity between 300 and 10 K. Compound **1** is not photosensitive with LEDs of wavelengths above 590 nm. *Bottom:* optical reflectivity spectra of **1** between 400 and 1000 nm at 300 K (left) to 10 K (right) showing the absence of photosensitivity at 590 nm and the weak efficiency at 505 nm. A spectroscopic white light of 0.08 mW cm^{-2} has been used for these measurements.

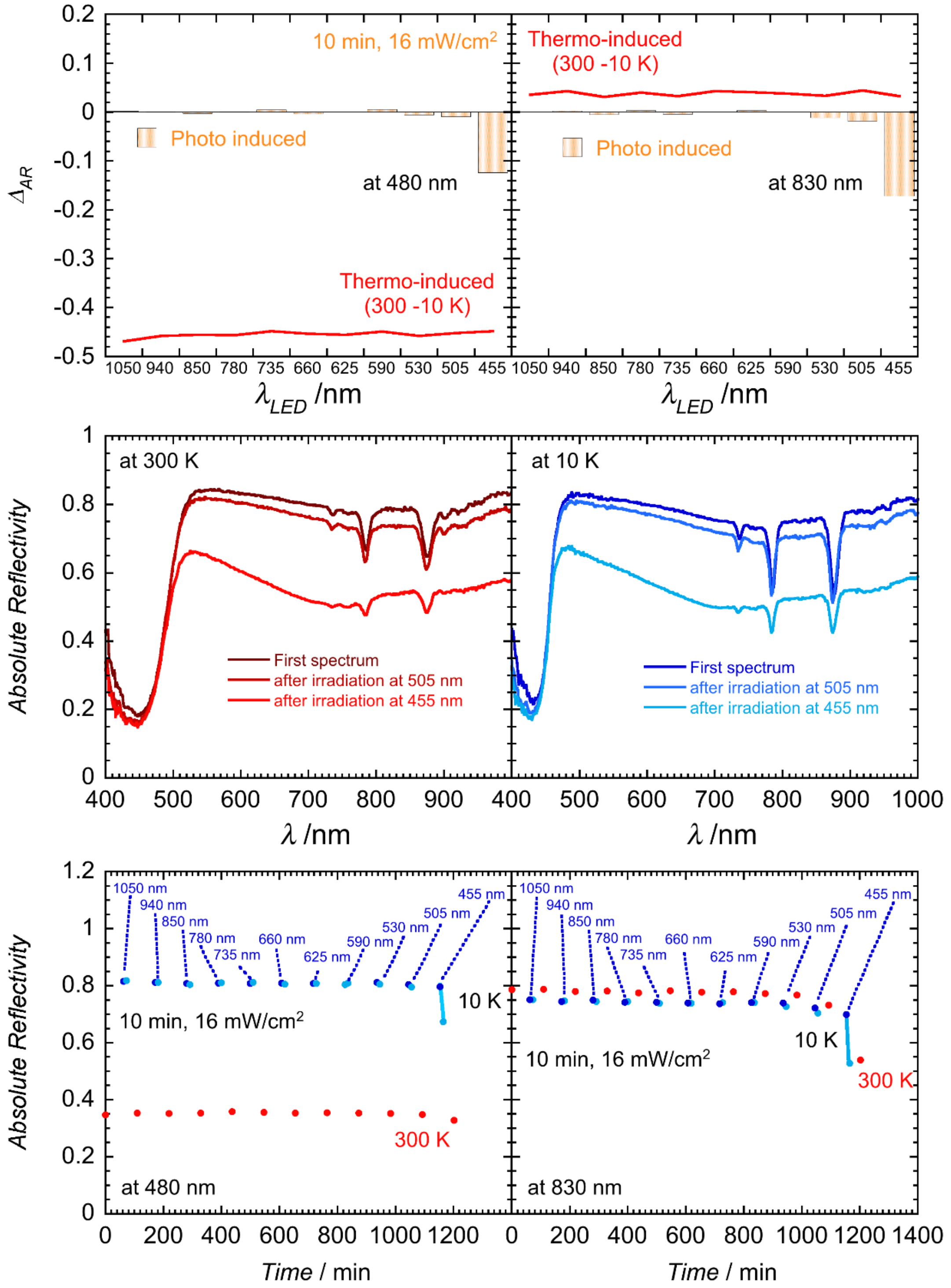

Figure S11 LED irradiations of **1** from 1050 to 455 nm. *Top:* after a fast cooling of **1** from 300 K in the dark, the plots show the variation of the absolute optical reflectivity at 10 K comparing before and after successive irradiations with different LEDs from 1050 nm to 455 nm ($\Delta_{AR} = AR_{after} - AR_{before}$; 10 minutes, at 16 mW cm^{-2}) recorded at 480 nm (left) and at 830 nm (right). Note that between each irradiation, the sample was heated to 300 K and cooled down to 10 K in the dark. For comparison, the red line is the thermal variation of the absolute optical reflectivity between 300 and 10 K. The compound **1** is not photosensitive with LEDs of wavelengths above 590 nm but becomes light sensitive for irradiation below. *Middle:* optical reflectivity spectra of **1** between 400 and 1000 nm at 300 K (left) to 10 K (right) showing the irreversible photosensitivity at 505 and 455 nm for comparison. *Bottom:* comparison of the 480 nm (left) and 830 nm (right) absolute reflectivity at 300 K (red dots), 10 K before (blue dots) and after (pale blue dots; 10 minutes, at 16 mW cm^{-2}) after successive irradiations at the indicated wavelength from 1050 to 455 nm. A spectroscopic white light of 0.08 mW cm^{-2} has been used for these measurements.

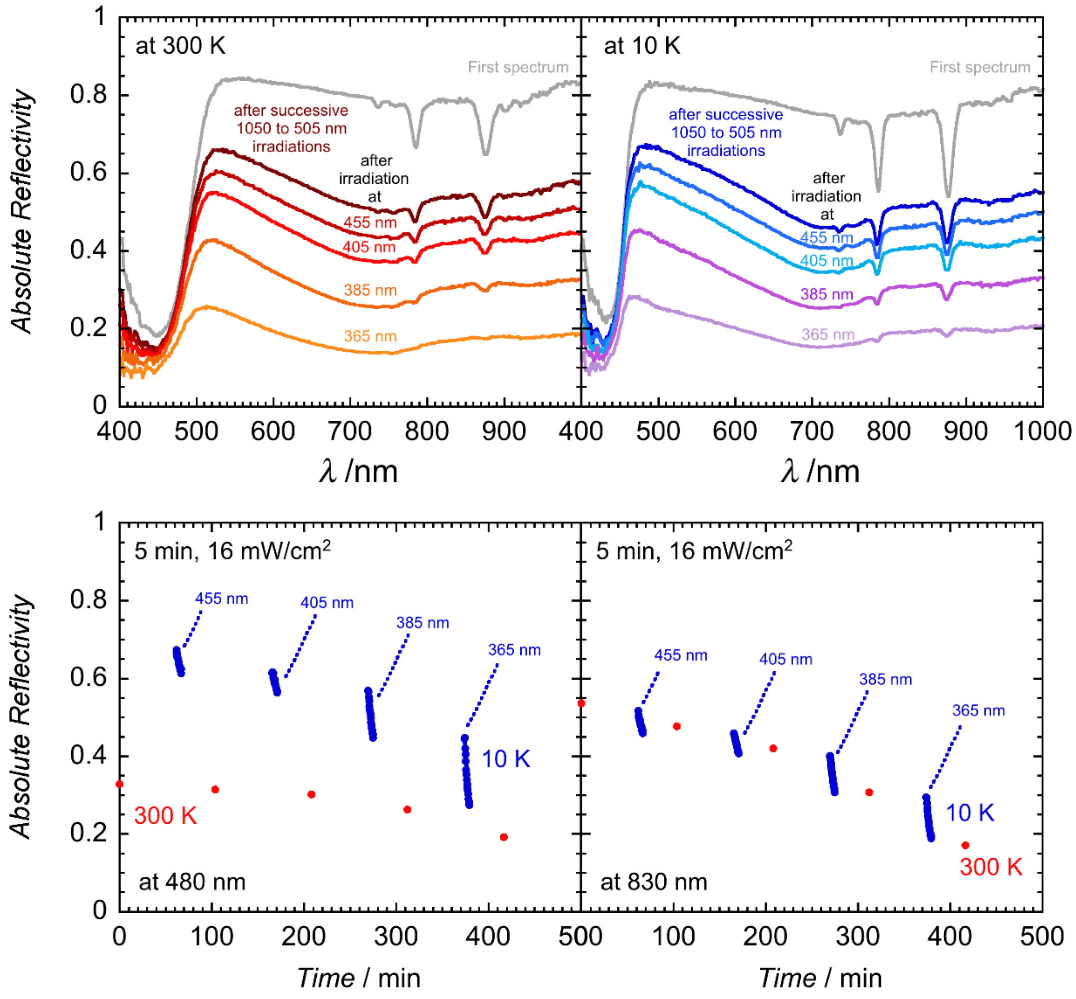

Figure S12 LED irradiation of **1** below 455 nm after the 1050 to 505 nm irradiations shown in Figure S10. *Top:* optical reflectivity spectra of **1** between 400 and 1000 nm at 300 K (left) to 10 K (right) showing the irreversible photosensitivity between 455 and 365 nm for comparison. *Bottom:* comparison of the 480 nm (left) and 830 nm (right) absolute reflectivity at 300 K (red dots), 10 K before (blue dots) and during (pale blue dots; 5 minutes, at 16 mW cm^{-2}) successive irradiations at the indicated wavelength from 455 to 365 nm. A spectroscopic white light of 0.08 mW cm^{-2} has been used for these measurements.

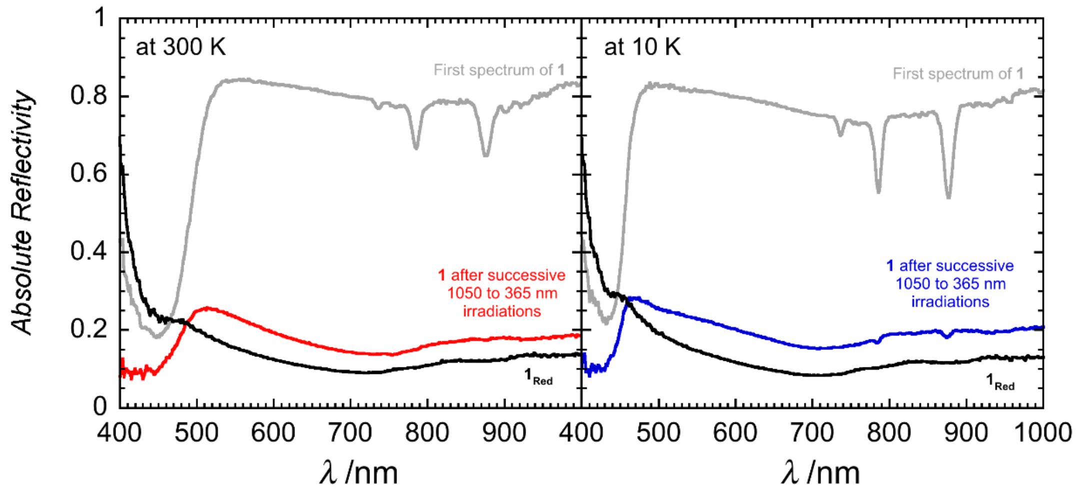

Figure S13 Optical reflectivity spectra of **1** and **1**$_{\mathbf{red}}$ between 400 and 1000 nm at 300 K (left) to 10 K (right) A spectroscopic white light of 0.08 mW cm^{-2} has been used for these measurements.

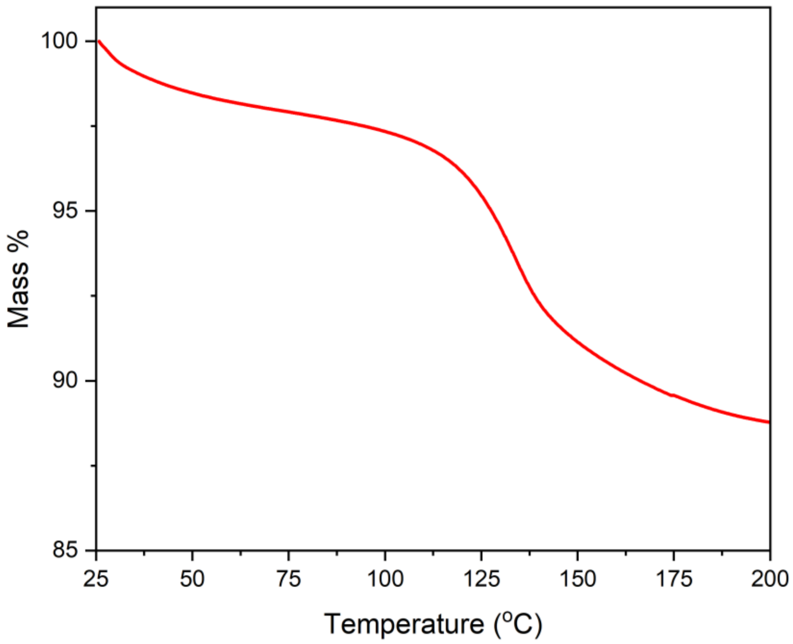

Figure S14 The thermo-gravimetric analysis curve of **1** from 25 – 200 °C. From the TGA, we observe a loss of solvent at room temperature which is attributed to a loss of Et_2O, consistent with the elemental analysis and equates to a loss of one Et_2O molecule and a 2% loss of mass. Above 100 °C, another loss of mass is observed, we attribute this to a loss of either THF or H_2O, which results in a loss of crystallinity.

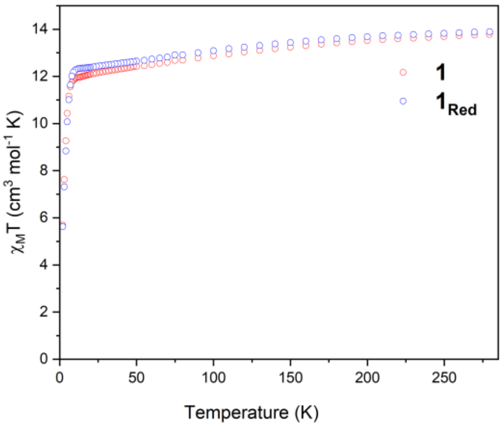

Figure S15 Temperature dependence of $\chi_M T$ from 280 to 2 K under an applied dc field of 1000 Oe for compounds **1** (red circles) and $\mathbf{1_{Red}}$ (blue circles).

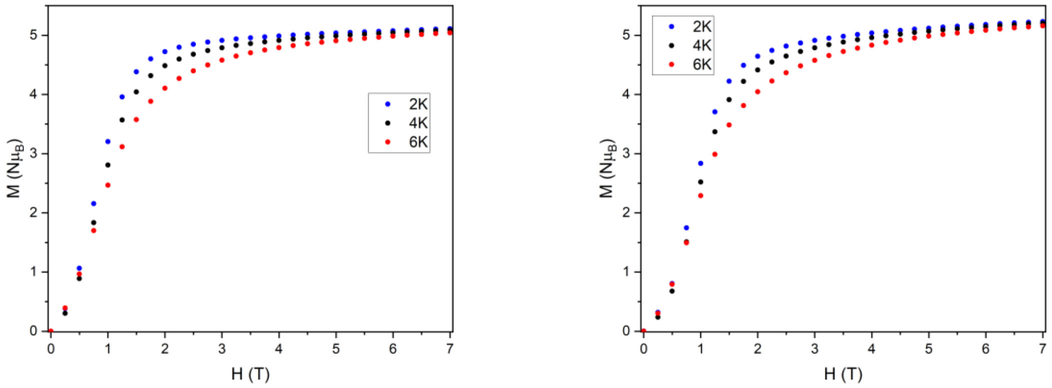

Figure S16 Magnetisation *vs*. field plots at 2, 4, 6 K from 0 – 7 T for **1** (left) and $\mathbf{1_{Red}}$ (right).

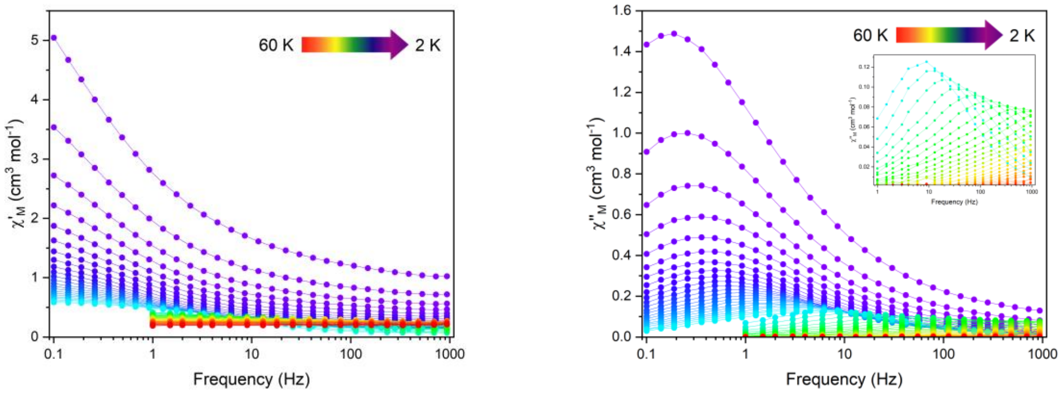

Figure S17 The frequency dependence of the in-phase (χ'_M; left) and out-of-phase (χ''_M; right) ac susceptibility signals of **1** in the temperature range 2 – 60 K under zero applied dc field. The out-of-phase magnetic susceptibility of **1** from 26 – 60 K (inset).

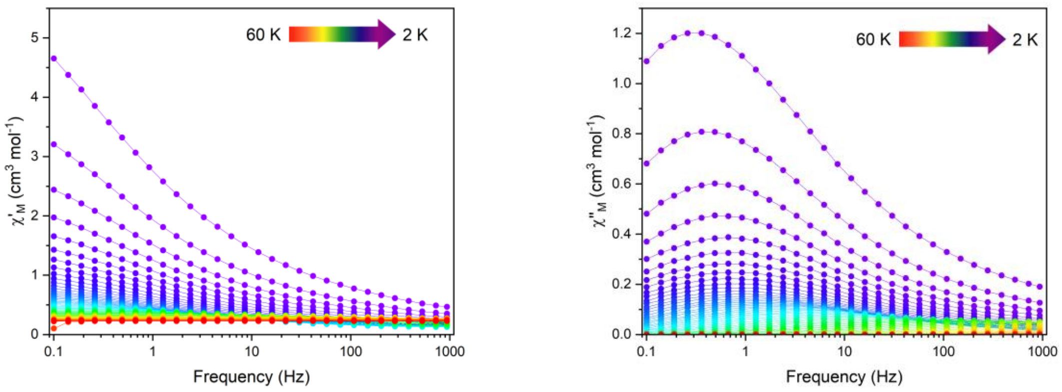

Figure S18 The frequency dependence of the in-phase (χ'_M; left) and out-of-phase (χ''_M; right) ac susceptibility signals of $\mathbf{1_{Red}}$ in the temperature range 2 – 60 K under zero applied dc field.

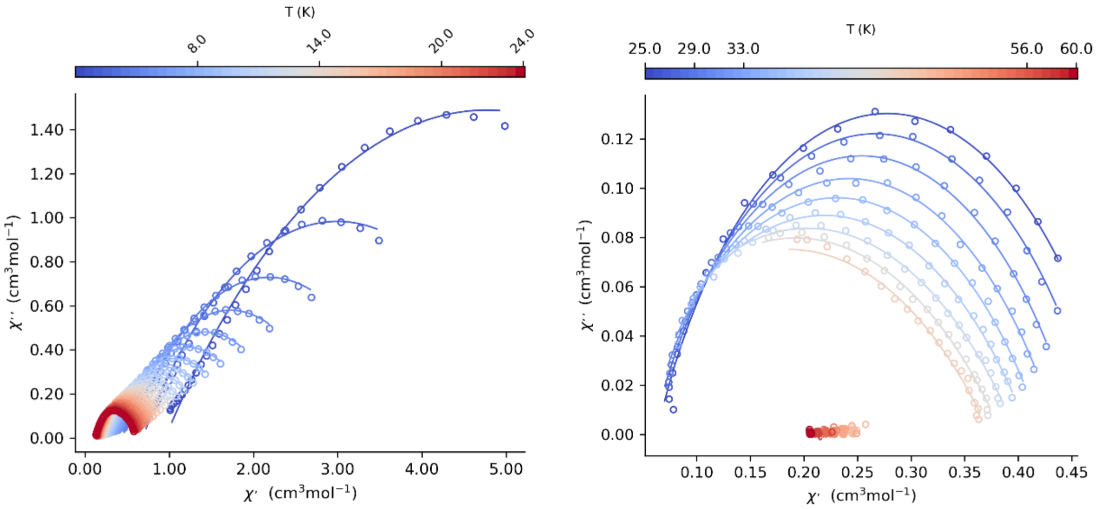

Figure S19 The Cole-Cole plot of in-phase (χ'_M) *vs.* out-of-phase (χ''_M) magnetic susceptibility of **1** in zero dc field from 2 – 24 K (left) and 25 – 33 K (right).

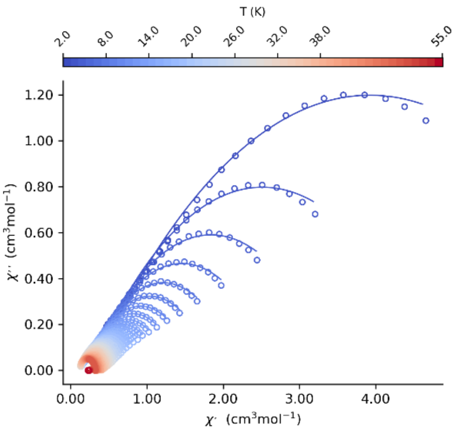

Figure S20 The Cole-Cole plot of in-phase (χ'_M) *vs.* out-of-phase (χ''_M) magnetic susceptibility in zero dc field for **1**$_{\mathbf{Red}}$ in zero dc field.

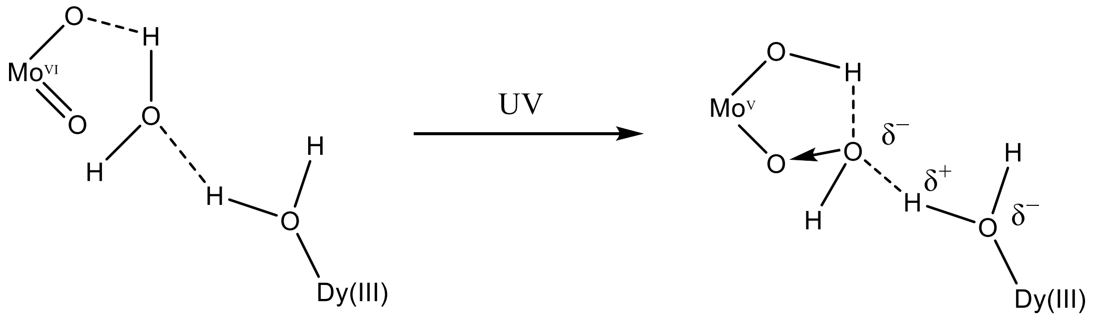

Figure S21 A schematic representation of the formation of a charge transfer complex in **1**$_{\mathbf{Red}}$ and the possible change in hydrogen bonding that may occur, causing an increased electron density in an equatorially coordinated water molecule to Dy(III).

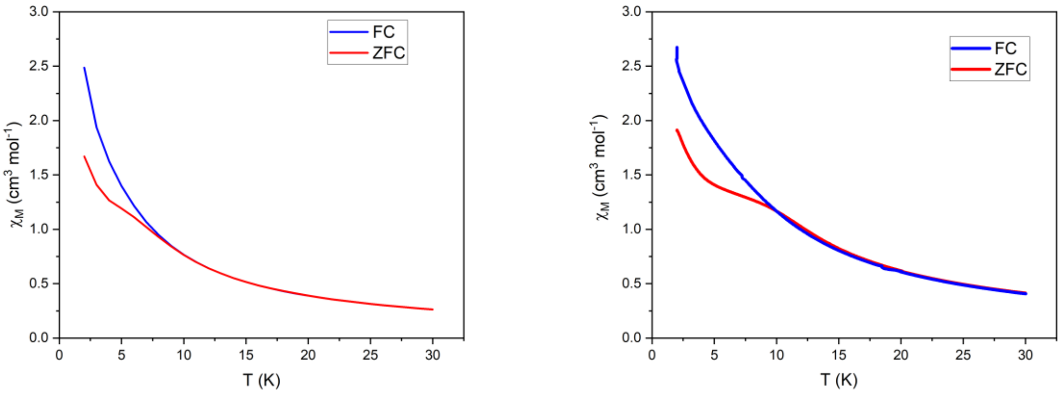

Figure S22 The field cooled (FC) and zero-field cooled (ZFC) magnetic susceptibility of **1** (left) and $\mathbf{1}_{\mathbf{Red}}$ (right) at 1000 Oe, with a sweep rate of 2 K/min, diverging at 8 K and 10 K, respectively.

To compare compounds **1** and **1$_{Red}$** to other *pseudo-D_{5h}* complexes, hysteresis measurements were also conducted at an increased sweep rate of 200 Oe/s, see below Figures S23 and S24:

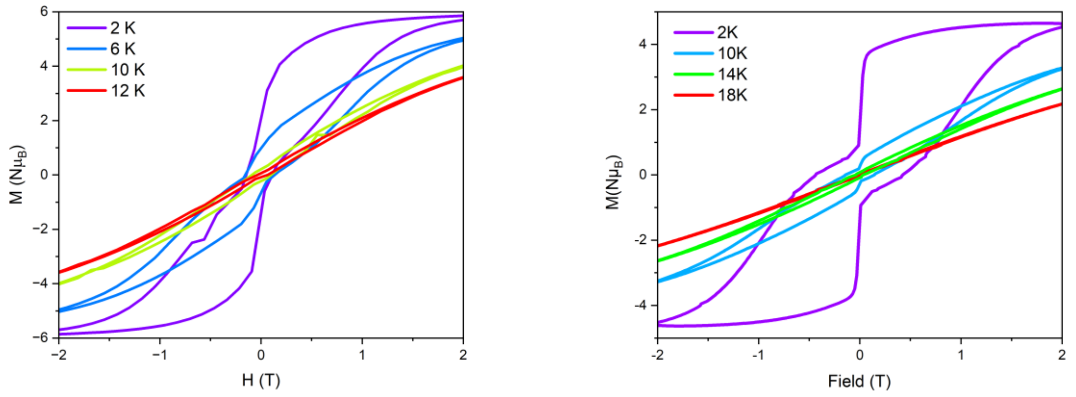

Figure S23 *M vs. H* hysteresis measurements for **1** (left) and **1$_{Red}$** (right), conducted with a sweep rate of 200 Oe/s.

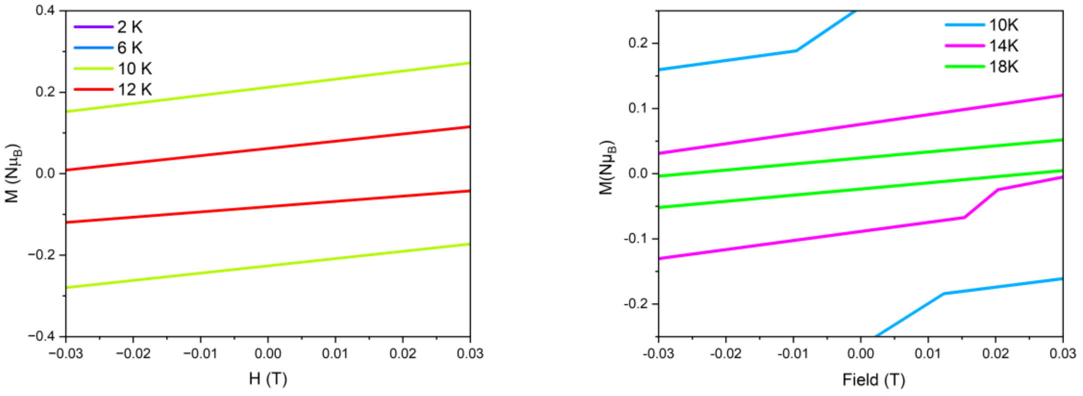

Figure S24 *M vs. H* hysteresis measurements for **1** (left) and **1$_{Red}$** (right), conducted with a sweep rate of 200 Oe/s, zoomed in at zero field.

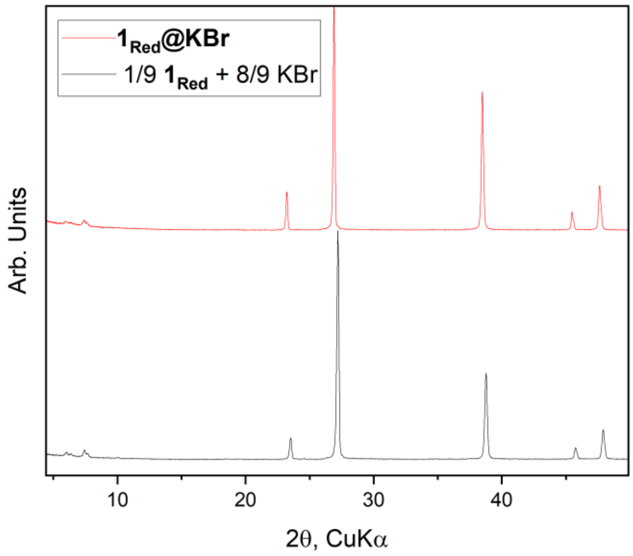

Figure S25 The powder X-ray diffraction patterns of $\mathbf{1_{Red}}$ and KBr and $\mathbf{1_{Red}}$**@KBr**, where the black line represents the powder pattern created from 1/9 of a normalised powder of $\mathbf{1_{Red}}$ and 8/9 of a normalised powder pattern of KBr. The red line represents the experimental powder pattern for $\mathbf{1_{Red}}$**@KBr**.

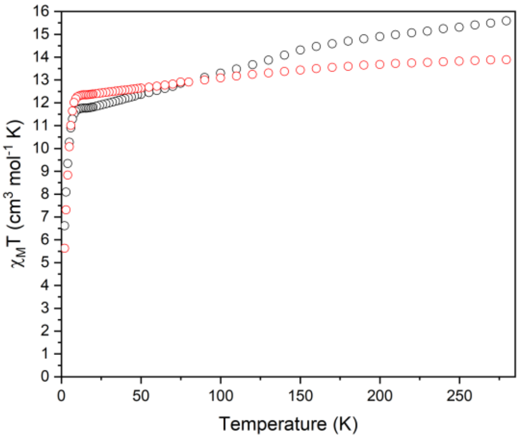

Figure S26 Temperature dependence of $\chi_M T$ from 280 to 2 K under an applied dc field of 1000 Oe for compound $\mathbf{1_{Red}}$**@KBr** (black) and $\mathbf{1_{Red}}$ (red).

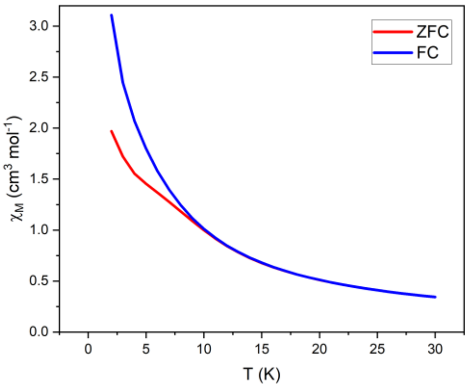

Figure S27 The field cooled (FC) and zero-field cooled (ZFC) magnetic susceptibility of $\mathbf{1_{Red}}$**@KBr** at 1000 Oe, with a sweep rate of 2 K/min, diverging at 10 K.

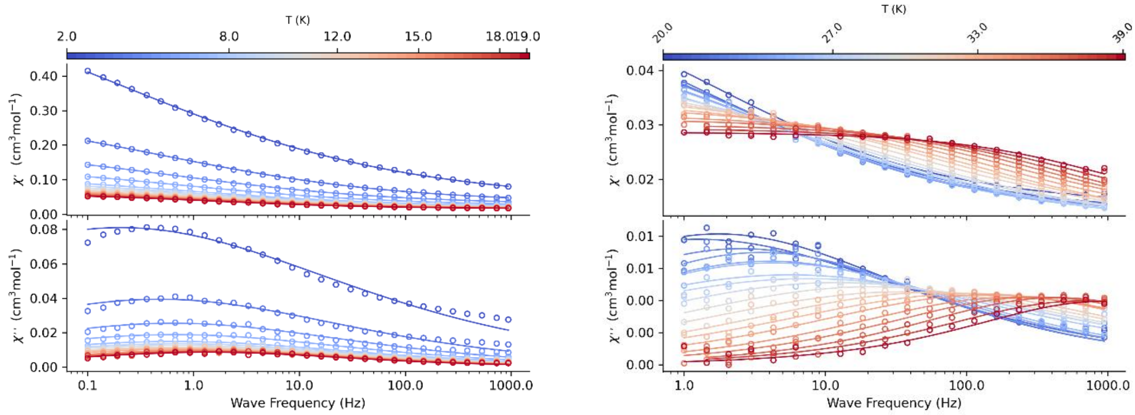

Figure S28 The frequency dependence of the in-phase (χ'_M) and out-of-phase (χ''_M) magnetic susceptibilities from 2 – 19 K (left) and 20 – 39 K (right) under zero applied dc field of $\mathbf{1_{Red}}$**@KBr**.

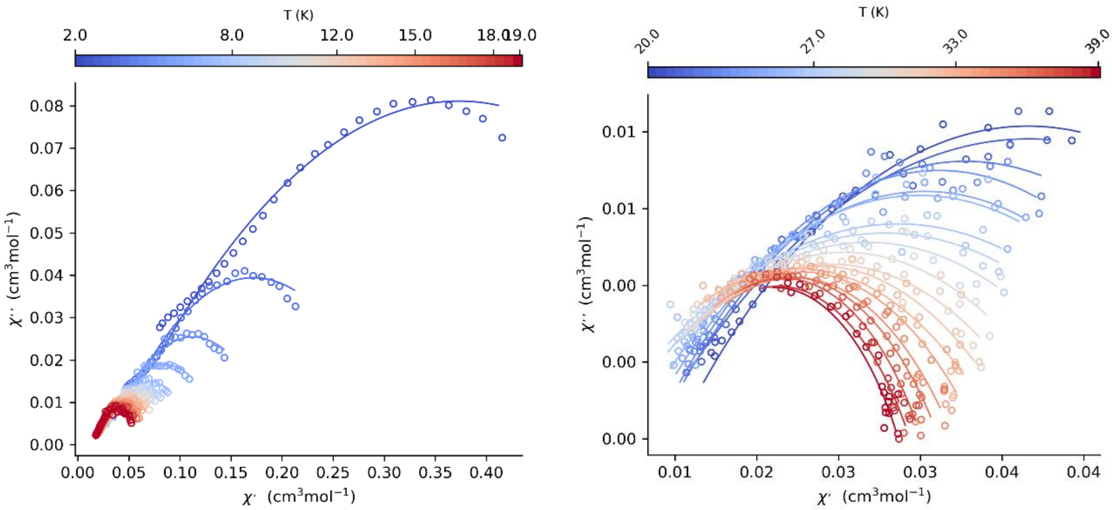

Figure S29 The Cole-Cole plot of in-phase (χ'_M) *vs.* out-of-phase (χ''_M) magnetic susceptibility of $\mathbf{1_{Red}}$**@KBr** in zero dc field from 2 – 19 K (left) and 20 – 39 K (right).

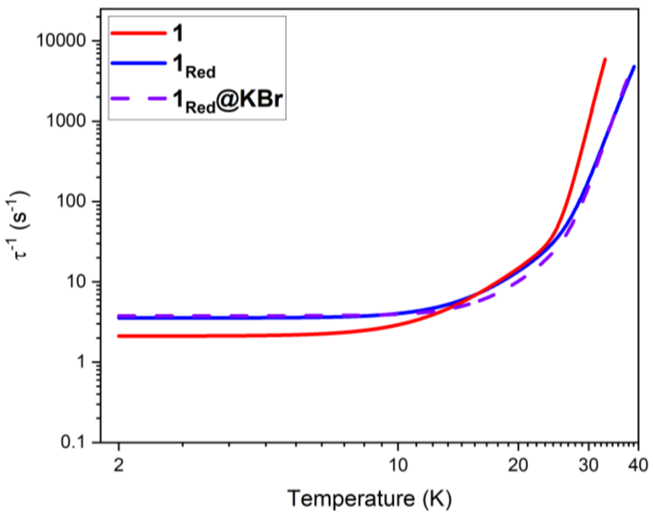

Figure S30 A comparison of the fits of the relaxation rates in compounds **1** (red), $\mathbf{1_{Red}}$ (blue) and $\mathbf{1_{Red}}$**@KBr** (purple dashed) under zero field.
